# Heritable Variation in Pea for Resistance Against a Root Rot Complex and Its Characterization by Amplicon Sequencing

**DOI:** 10.3389/fpls.2020.542153

**Published:** 2020-11-03

**Authors:** Lukas Wille, Monika M. Messmer, Natacha Bodenhausen, Bruno Studer, Pierre Hohmann

**Affiliations:** ^1^Department of Crop Sciences, Research Institute of Organic Agriculture (FiBL), Frick, Switzerland; ^2^Molecular Plant Breeding, Institute of Agricultural Sciences, ETH Zurich, Zurich, Switzerland

**Keywords:** damping-off, disease tolerance, foot rot, legumes, plant-microbe interactions, resistance breeding, root rot

## Abstract

Soil-borne pathogens cause severe root rot of pea (*Pisum sativum* L.) and are a major constraint to pea cultivation worldwide. Resistance against individual pathogen species is often ineffective in the field where multiple pathogens form a pea root rot complex (PRRC) and conjointly infect pea plants. On the other hand, various beneficial plant-microbe interactions are known that offer opportunities to strengthen plant health. To account for the whole rhizosphere microbiome in the assessment of root rot resistance in pea, an infested soil-based resistance screening assay was established. The infested soil originated from a field that showed severe pea root rot in the past. Initially, amplicon sequencing was employed to characterize the fungal microbiome of diseased pea roots grown in the infested soil. The amplicon sequencing evidenced a diverse fungal community in the roots including pea pathogens *Fusarium oxysporum, F. solani*, *Didymella* sp., and *Rhizoctonia solani* and antagonists such as *Clonostachys rosea* and several mycorrhizal species. The screening system allowed for a reproducible assessment of disease parameters among 261 pea cultivars, breeding lines, and landraces grown for 21 days under controlled conditions. A sterile soil control treatment was used to calculate relative shoot and root biomass in order to compare growth performance of pea lines with highly different growth morphologies. Broad sense heritability was calculated from linear mixed model estimated variance components for all traits. Emergence on the infested soil showed high (*H*^2^ = 0.89), root rot index (*H*^2^ = 0.43), and relative shoot dry weight (*H*^2^ = 0.51) medium heritability. The resistance screening allowed for a reproducible distinction between PRRC susceptible and resistant pea lines. The combined assessment of root rot index and relative shoot dry weight allowed to identify resistant (low root rot index) and tolerant pea lines (low relative shoot dry weight at moderate to high root rot index). We conclude that relative shoot dry weight is a valuable trait to select disease tolerant pea lines. Subsequently, the resistance ranking was verified in an on-farm experiment with a subset of pea lines. We found a significant correlation (*r*_*s*_ = 0.73, *p* = 0.03) between the controlled conditions and the resistance ranking in a field with high PRRC infestation. The screening system allows to predict PRRC resistance for a given field site and offers a tool for selection at the seedling stage in breeding nurseries. Using the complexity of the infested field soil, the screening system provides opportunities to study plant resistance in the light of diverse plant-microbe interactions occurring in the rhizosphere.

## Introduction

Pea (*Pisum sativum* L.) is an important protein sources for human consumption and animal feed. It has an annual worldwide production of 36 mega tons, making it the second most important pulse after common bean (FAO, 2019). Pea represents a valuable crop for sustainable cropping systems. Through the symbiosis with nitrogen-fixing rhizobacteria, pea cultivation improves soil fertility and reduces the demand for external nitrogen fertilizers (Foyer et al., 2016). In Europe, an increase in pea acreage is expected due to current incentives to promote regionally produced plant-based protein as an alternative to overseas soybean imports (Reckling et al., 2016).

Pea cultivation is challenged by various abiotic and biotic stresses (Rubiales and Mikic, 2014). Most importantly, several soil-borne diseases threaten pea cultivation. The most devastating diseases are caused by fungal pathogens, including various species of the genus *Fusarium* (most notably, *F. solani*, *F. avenaceum*, and *F. oxysporum*), *Didymella pinodes* (formerly known as *Mycosphaerella pinodes*), *D. pinodella* (formerly known as *Phoma medicaginis* var. *pinodella*), *Rhizoctonia solani, Sclerotinia sclerotiorum*, and the oomycetes *Aphanomyces euteiches* and *Pythium* spp. (Wille et al., 2019). These pathogens are responsible for severe seed rot, damping-off, seedling blight, and root and foot rot.

Multiple pea pathogen species co-occur in the field, leading to the adoption of the term pea root rot complex (PRRC). It has repeatedly been shown that root-infecting pathogens interact and aggravate disease in pea (Kerr, 1963; Muehlbauer and Kraft, 1973; Shehata et al., 1983). However, research has only recently readopted this line of work in order to understand the complexity, distribution and interplay among multiple pathogens in PRRC (Šišić et al., 2017; Taheri et al., 2017; Willsey et al., 2018). Moreover, the rhizosphere harbors a vast diversity of micro-organisms involved in plant-microbe and microbe-microbe interactions, ranging from plant pathogenic to plant beneficial and antagonistic to synergistic, respectively (Müller et al., 2016).

Microbial dysbiosis caused by inappropriate culturing practices such as narrow crop rotations are often at the origin of outbreaks of soil-borne diseases. For instance, it has been shown that increasing the frequency of pea in a 4-years crop rotation causes the build-up of fungal pathogens in the soil (Bainard et al., 2017), and that pathogenic species displace beneficial fungi in diseased pea roots (Xu et al., 2012). Respecting long rotation breaks is the most constructive strategy in this regard. However, this is in conflict with efforts to increase the cultivation area of legume crops in general and pea in particular. Control by chemical fungicides is only available through the application of seed treatments, but shows only moderate effects on emergence and disease severity of pea in infested fields (Xue, 2003b; Wu et al., 2019). Biological control agents are a possible alternative to chemicals, however, they still need to demonstrate their efficacy to confer protection under field conditions (Alabouvette et al., 2009).

Breeding resistant varieties is considered the most promising approach for sustainable pea cultivation, especially with the increasing necessity to shift from large-scale applications of chemical pesticides and seed treatments toward more integrative solutions (Rubiales et al., 2015). However, multipartite interactions among pathogens and other microbes in the PRRC are rarely considered in resistance studies. Resistance screenings are commonly performed under controlled conditions, where seedlings, grown on sterile substrate, are inoculated with single pathogen isolates. This practice allows for reproducible mono-factorial disease scorings and has led to the identification of resistance sources for major pathogens in various pea germplasm collections over the last decades (Infantino et al., 2006; Rubiales et al., 2015). However, resistance against individual pathogen species or strains assessed under controlled conditions is frequently ineffective when moved to the field as different pathogens are present in the PRRC (Hamon et al., 2011). The work of Abdullah et al. (2017) suggests that plant-pathogen interactions and resistance should be studied in field representative systems to achieve progress in disease management.

The observation that multiple soil-borne pathogens interact to shape the development of root rot is underlined by results from field resistance trials. For instance, pea breeding lines exhibited different levels of resistance to Aphanomyces root rot when evaluated at two different locations in the north-west U.S. (Weeden et al., 2000). Further, Hamon et al. (2011) reported significant genotype-by-environment interactions in a quantitative trait loci (QTL) study for Aphanomyces root rot resistance carried out in different French and United States field sites. The authors of these studies concluded that different co-occurrence patterns of pathogens at different field sites explain site-specific resistance rankings and identified resistance loci. These results highlight the importance of respecting the soil microbial community as an integral part of the environment.

Plant breeders rely on reproducible screening systems that allow the screening of large numbers of lines. These screening systems need to include major factors of the target environment—including the soil type and the microbiome composition of that particular soil—to provide reliable and field-relevant data for subsequent breeding efforts (Duc et al., 2015; Wei and Jousset, 2017). It is widely accepted that plant health also depends on the plant-associated microbial community (Berg et al., 2017). Thus, incorporating microbiome-associated phenotypes in resistance breeding will provide a more solid basis to breed crops for enhanced disease resistance (Oyserman et al., 2018; Wille et al., 2019).

In order to account for the interactions between the plant genotype and the pathogen complex embedded in the entire rhizosphere microbiome, we designed a resistance screen based on infested soil. The overall aim of this study was to develop an infested soil-based resistance screening at seedling stage under controlled conditions to allow for a reproducible assessment of resistance against PRRC and to assess its relation to resistance in the field. Specifically, we aimed at (i) establishing a screening system that allows to differentiate between susceptible and resistant pea lines; (ii) assessing broad-sense heritability of various disease-related traits; (iii) examining the relationship among these traits in order to better understand the disease expression and identify most suited parameters to assess PRRC resistance; and (iv) relating the controlled conditions resistance ranking with field performance in order to evaluate the relevance of the proposed screening tool for resistance breeding. In addition, we applied amplicon sequencing to characterize fungal diversity present in the rhizosphere of PRRC diseased pea to identify potential pathogens and beneficials.

## Materials and Methods

### Plant Material

This study is based on a set of 261 pea (*Pisum sativum*, L.) lines, including 177 genebank accessions from the USDA-ARS GRIN Pea Core Collection^1^, 47 advanced breeding lines provided by a private organic breeder organization (Getreidezüchtung Peter Kunz, Switzerland)^2^ and 34 registered cultivars from Europe (Supplementary Table 1). Additionally, two cultivars were included as reference lines, namely “EFB.33” (experimental identifier: “C1”), a cultivar with known resistance capacities against root rot (Baćanović-Šišić et al., 2018), and “Respect” (”C2”), a standard registered variety susceptible to root rot. The set contained full-leaf and semi-leafless type pea lines. All pea lines were multiplied by a commercial seed company (Sativa Rheinau, Switzerland) at one field location prior to the experiment.

### Infested Field Soil Used for Controlled Conditions Screening

Naturally infested soil was collected from a field under certified organic production located in Kirchlindach, Canton Bern, Switzerland (47°00′14.5″N 7°24′37.7″E) in March 2016. Physico-chemical soil characteristics are given in Supplementary Table 2. Soil from this field site was previously assessed in a study on PRRC in Swiss and German fields and showed strong signs of PRRC (Fuchs et al., 2014). Sieved soil was stored in polypropylene boxes at 4°C in the dark until further use. Subsequent experiments with the same batch of soil have repeatedly confirmed that the pathogenicity of the soil persist over time.

### Protocol of Controlled Conditions Resistance Screening Based on Infested Soil

The set of pea lines was evaluated for resistance against PRRC in the naturally infested soil. Seeds were surface-sterilized in 70% ethanol for 30 s followed by a 1:1 (v:v) ddH_2_O-bleach solution (M-Classic Javel Wasser, Migros, Switzerland; final concentration ∼ 2.5%) for 10 mins. Finally, seeds were thoroughly rinsed in ddH_2_O and soaked for 2 h. Four seeds per line were planted in a 2:1 (v:v) mixture of infested soil and sterilized sand (Quartz d’Alsace, 0.2–0.63 mm grain) in plastic pots (200 ml, Migros, Switzerland). For the control treatment, soil, and sand were sterilized (X-Ray irradiation 30–100 kGy, Synergy Health Däniken AG, Switzerland) and kept vacuum packed at 4°C in the dark until use.

Pots were arranged in a randomized complete block design with four replications. The replications were run in a series over 4 months. Complete blocks were further divided into five incomplete blocks of 52 or 53 pea lines augmented with two entries of the two reference cultivars C1 and C2. Each experimental unit was set up as a pair of two pots, containing either infested soil or sterilized soil. The five incomplete blocks of one replication were sown on five consecutive days and harvested over 5 days in the same order. Plants were grown under controlled conditions in the growth chamber for 21 days. A 16/8 light/dark cycle was applied, providing a photosynthetically active photon flux density of 200 μmol m^–2^ s^–1^ over the waveband 400–700 nm. Plants were watered with tap water every 72 h by flooding the pots 4 cm high for 30 min. Growth chamber mean (± SE), minimum and maximum temperature over the course of the experiment was 20.1 ± 0.3°C, 17.7 ± 0.7°C, and 26.9 ± 2.9°C, respectively, and mean, minimum and maximum relative humidity was 85.3 ± 14.9%, 40.4 ± 6.9%, and 94.6 ± 12.2%, respectively. Pots were inspected on a daily basis for seedling emergence and plants were thinned out to reach a maximum of three plants per pot. Plants emerging after 14 days were removed and not considered in any analysis.

### Phenotypic Assessments in the Controlled Conditions Resistance Screening

Seedling emergence was recorded 14 days after sowing and a plant emergence rate (n/4; 0–1) was calculated on a per pot basis. Twenty-one days after sowing, the plants were removed from the pots and roots were washed under running tap water. Plants were visually inspected and the following disease scores and vitality parameters were assigned to individual plants: (1) *Controlled Conditions Root Rot Index* (RRI*_*CC*_*: 1 = no symptoms − 6 = complete disintegration of the root system; Supplementary Figure 2). This RRI*_*CC*_* was developed as the observed disease picture did not fit previously described disease score indexes for major pea root rot pathogens such as *Fusarium solani* (Grünwald et al., 2003; Bodah et al., 2016), *Fusarium* ssp. and *Didymella pinodella* (Pflughöft et al., 2012) or *Aphanomyces euteiches* (Moussart et al., 2008); (2) *Cortex Decay Index* (CDI: 1 = no symptoms − 5 = total disintegration of cortex); (3) *Shoot Lesion Index* (SLI: 1 = no symptoms on the epicotyl − 6 = discoloration and disintegration of stem base); (4) *Nodulation Index* (NOD: 1 = no nodules − 7 > 60 nodules). A full description of traits 1–4 is given in Supplementary Table 3. Pot medians were calculated from scores of individual plants for these four traits. Furthermore, (5) *Plant height* (from the cotyledons to youngest node); and (6) *Disease Progress* (DIS; length of lesion on the stem above the cotyledons) were measured in (cm); and (7) a *Wilted Nodes Ratio* (WIL; N*_*Wilted nodes*_* /N*_*To*__*tal nodes*_*)*_*Infested soil*_* − (N*_*wilted nodes*_*/N*_*To*__*tal nodes*_*)*_*Sterile soil*_*) was calculated. Pot means were calculated for these three traits. Finally, fresh shoot and root biomass was recorded. Subsequently plants were dried at 105°C until constant weight before recording dry weight. Biomass measurements per pot were standardized with the number of plants per pot at harvest. *Relative Shoot and Root Fresh and Dry weights* were calculated by dividing the biomass of the infested soil treatment by the biomass of the corresponding sterile control treatment of the same genotype in each replication (SFW*_*Rel.*_*, RFW*_*Rel.*_*, SDW*_*Rel*_*,. and RDW*_*Rel.*_*, respectively).

### On-Farm Verification of the Controlled Conditions Resistance Screening

Seven pea lines were selected from the controlled conditions experiment and evaluated together with the two reference cultivars on two different on-farm sites in 2018 in order to evaluate the field predictability of the established screening. The pea lines were selected based on contrasting root rot resistance (RRI*_*CC*_*) and to represent gene bank accessions as well as breeding lines. One experimental site was located in the field where the naturally infested soil for the pot trial was obtained (“heavily infested site”). The second site was located within 50 m to the first site (“moderately infested site”), with similar soil characteristics compared to the heavily infested site (Supplementary Table 2). According to the farmer, this second site was less affected by pea root rot in 2014, when both sites were sown with a pea/barley mixture and managed in the same way. The crop rotation for both field sites from 2014 to 2017 was: Pea/barley—winter wheat—oat/vetch—potato—winter wheat. Field sites were on-land plowed and the seed bed preparation was carried out with a spring-tooth harrow. Both sites were arranged in a randomized complete block design with three replications per pea line. The plot size was 1.7 m × 1.5 m with three single rows of pea flanked by a row of spring barley (“Atrika”) on each side. Pea and barley seeds were sown on April 10, 2018 at a density of 94 and 200 seeds per m^2^, respectively. Sowing depth was 5 cm. The trial was operated under certified organic farming conditions. Weeding was done manually as needed. Twenty-four days after sowing “Kaolin” (Surround, Stähler) was applied according to manufacturer instruction (32 kg/ha) to combat pea leaf weevil (*Sitona lineatus*). Cumulative rainfall March–April–May 2018 in the region (*Agrometeo* station “*Oeschberg*”) was 143.3 mm (2008–2017 long-term mean ± SE for the same period: 312.0 ± 44.3 mm).

Fifty-five days after sowing, 15 plants per plot were randomly selected, carefully dug out and roots were washed with tap water. As for the RRI*_*CC*_*, the time point was selected based on a good differentiation of the disease expression among the pea lines. In the field, plant and disease development was slower than in controlled conditions. Root rot was assessed using a 1–8 scoring scale adapted from Pflughöft (2008) to assess adult plant disease symptoms under field conditions (1 = no symptoms, 2 = small localized lesions at hypo-/epicotyl, 3 = light-brown discoloration/lesion, with < 50% circumference of the tap root, 4 = dark discoloration/lesion, with > 50% circumference of the tap root, 5 = progress of the discoloration up to first lower leaf and/or < 3 cm in the tap root, possibly localized drying and bursting, 6 = progress of the discoloration further than first lower leaf and/or > 3cm in the tap root, possibly localized drying and bursting, 7 = decay of root and/or lower stem cortex, possibly visible vascular tissues, 8 = total disintegration of the root system or the stem, plant dying. A field root rot index (RRI*_*Field*_*) per plot was calculated as the median of 15 plants.

### Statistical Analysis of Phenotypic Data

All calculations were performed with R 3.5.2 (R Core Team, 2018). Mixed model analyses were performed on the controlled conditions screening data with the following model: *y*_*ijk*_ = *μ* + *g*_*i*_ + *r*_*j*_ + *s*_*k*_ + *ε_*ijk*__._* Here, *y*_*ijk*_ represents the pot observation for trait *y*, *μ* denotes the overall mean, *g*_*i*_ the effect of pea line *i*, *r*_*j*_ the effect of replicate *j*, *s*_*k*_ the effect of the incomplete block *k* and *ε_*ijk*_* the residual. For the estimation of genotypic means, the effects μ, *g*_*i*_ and *r*_*j*_ were considered fixed, the others as random. In order to meet the assumptions of the model plant height and biomass weights were log_10_-transformed before analysis. Relative biomass, wilted nodes ratio, disease progress, root rot, cortex decay, shoot lesion, and nodulation indices were transformed using an inverse Lambert W x *F*_*X*_ function before analysis (*LambertW* package) (Goerg, 2015). Data on seedling emergence was analyzed as probabilities using a generalized linear mixed model fitting a binomial distribution of the errors using maximum likelihood estimation. A Wald χ^2^-test with type II sums of squares (*Anova* function in the *car* package) was applied on each model to calculate the *p*-values for the fixed effects of pea line and replicate. Marginal *R*^2^ were calculated according to Nakagawa et al. (2013).

For the estimation of variance components due to genotypic effects of pea lines (*σ^2^_*g*_*) and residual factors (σ^2^_ε_), *g*_*i*_ was considered as random. Variance components were computed by restricted maximum likelihood, except for seedling emergence, where a maximum likelihood approach was used. Broad sense heritability (*H*^2^) on entry mean basis were calculated as: *H^2^* = *σ^2^_*g*_*/(*σ^2^_*g*_*+σ^2^_ε_/R), where *σ^2^_*g*_* is the genetic variance component, *σ^2^_ε_* the residual variance component and R the number of replicates. Bootstrapping (*bootMer* function in the *lme4* package) was used to estimate standard errors of variance components and *H*^2^. Mixed model calculations were done using the packages *lme4* 1.1–20 (Bates et al., 2015) and *emmeans* (Lenth, 2019). Compliance of the model assumptions was controlled by visual inspection of the residual plots.

A Principal Component Analysis (PCA) was performed on a subset of 11 phenotypic variables, using the estimated means from the mixed model analysis (*PCA* function in the *FactoMineR* package (Le et al., 2008)).

Pairwise relationships between the estimates of plant emergence, root rot index and relative shoot dry weight were explored by calculating Spearman’s correlation coefficients (*cor.test* function).

A linear fixed effect model was used to analyze the root rot data from the on-farm trial (RRI*_*Field*_*): *y*_*ijk*_ = *μ* + *g*_*i*_ + *f*_*j*_ + *gf*_*ij*_ + *b*_*k*_ + *ε_*ijk*_*, where *y*_*ijk*_ represents the plot observation for RRI*_*Field*_*, *μ* denotes the overall mean, *g*_*i*_ the effect of pea genotype *i*, *f*_*j*_ the effect of field site *j*, *gf*_*ij*_ the effect of the interaction between genotype *i* and field site *j*, *b*_*k*_ the effect of block *k* and *ε_*ijk*_* the residual. As for the controlled conditions experiment, root rot data was transformed using an inverse Lambert W x *F*_*X*_ function before analysis. Spearman’s correlation coefficients were calculated for the analysis of pairwise relationships between SDW*_*Rel.*_*, RRI*_*CC*_*, and RRI*_*Field*_*. All data is presented as the back-transformed means.

### Assessment of Root Fungal Community Composition

In order to describe the fungal community composition present in the infested field soil and in diseased pea roots, lines S12 and S164 were randomly selected from our pea collection. Both lines are full leaf type, S12 with pigmented flowers and S164 with withe flowers. Both lines were grown in four replications in infested soil as described above in addition to the screening experiment. Diseased roots and rhizosphere soil were sampled after 21 days according to Lundberg et al. (2013). Briefly, roots were shaken to remove loosely attached soil and washed in sterile 50 ml tubes in 25 ml sterile water by vortexing. Washed roots were stored at −20°C until further processing. The rhizosphere soil suspension was centrifuged for 15 min at 3,200 g. The pellet was resuspended, transferred to 1.5 ml tubes and centrifuged again at 10,000 g for 5 min to form a pellet. DNA was extracted from 1 g lyophilised rhizosphere soil and 15 mg roots using the NucleoSpin Soil Kit (Macherey-Nagel) and the Quick-DNA Plant/Seed Miniprep Kit (Zymo Research), respectively, according to manufacturer’s instructions. DNA was quantified using QUBIT DNA BR assay (Thermo Fisher Scientific). Primers ITS1f (Gardes and Bruns, 1993) and ITS2 (White et al., 1990) were used to amplify the ITS1 region. These primers were selected based on preliminary tests, showing the least amplification of plant DNA. The reaction volume was 20 μl and contained 1x 5 Prime Hot Master mix, 200 nM of each primer and 1ng of template DNA. The PCR program consisted of an initial denaturation step of 2 min at 95°C, followed by 32 cycles (based on qPCR pre-test) of denaturation at 94°C for 20 sec, annealing at 52°C (based on gradient PCR pre-test) for 10 sec, elongation at 65°C for 50 s followed by a final elongation step at 65°C for 10 min. PCR reactions were run in triplicates with a negative control. Triplicate PCR products were pooled and purified with home-made solid-phase reversible immobilization (SPRI) beads^3^. Pooled PCR products were indexed using the Nextera XT Index kit v2 (Illumina) with 1x KAPA HiFi HotStart ReadyMix according to manufacturer’s instructions, verified on 1.5% agarose gel, purified using SPRI beads, quantified using QUBIT DNA BR assay (Thermo Fisher Scientific) with SPARK 10M Platereader (Tecan) and combined in equimolar fashion. The library was quantified and quality was validated with Tapestation 2200 (Agilent Technologies): The final average amplicon length was ∼400–500 bp. The library was sequenced at the Genomic Diversity Center (Zurich, Switzerland) on the Illumina MiSeq Personal Sequencer (Illumina) using a 600 cycle v3 Sequencing kit, in paired-end 2 × 300 bp mode. The raw sequencing data is available from the European Nucleotide Archive under the study accession PRJEB39076.

The MiSeq data was processed similar to the workflow described in Bodenhausen et al. (2019) (Supplementary Table 4). Briefly, read ends were trimmed using *usearch* v10.0.240 (Edgar, 2010) and subsequently merged into amplicons using *FLASH* v1.2.11 (Magoc and Salzberg, 2011). In a next step, *CUTADAPT* v1.12 was used to trim off primer sequence (Martin, 2011). Subsequently, reads were quality filtered using *prinseq-lite* v0.20.4 (Schmieder and Edwards, 2011). The quality filtered sequences were clustered into operational taxonomic units (UPARSE-OTUs, ≥ 97% sequence similarity) and amplicon sequence variants (UNOISE) using *usearch* v10.0.240 (Edgar, 2010). *SINTAX* (Edgar, 2016) was used for taxonomic assignments using *UNITE* v7.2 (Abarenkov et al., 2010) database for the fungal community. Taxonomic information of unassigned sequences (below family rank) were further explored using BLAST analysis of the Nucleotide collection database. BLAST taxonomic information was considered at query cover > 92% and sequence identity of 100%.

Statistical analyses were performed using R 3.5.2 (R Core Team, 2018). Permutational multivariate analysis of variance (PERMANOVA) was performed to determine the effects of the factor levels bulk soil, rhizosphere soil and roots. PERMANOVA analyses of the fungal community compositions revealed no differences between the two pea genotypes for the rhizosphere and the root (data not shown). Therefore, relative OTU abundances in the rhizosphere and root of pea are analyzed based on the mean of both genotypes. Root-enriched OTUs were identified using *EdgeR* (Robinson et al., 2010). The data was filtered to remove low-abundant OTUs (OTUs with less than four sequences in less than four samples) and normalized by trimmed mean of *M*-values normalization (Robinson and Oshlack, 2010). Dispersion was estimated with the *estimateGLMRobustDisp* function (Zhou et al., 2014). A negative binomial model was fitted to the data with the *glmFit* function and the coefficient of interest was tested with the *glmLRT function*. To calculate the mean relative abundance, the data were first transformed by dividing each count by the total sum and transformed with log_2_(x+1). Data is presented as the back-transformed means (x̄_*bt*_ = 2^ȳ−1).

## Results

### Assessment of the Controlled Conditions Resistance Screening

**FIGURE 1:**
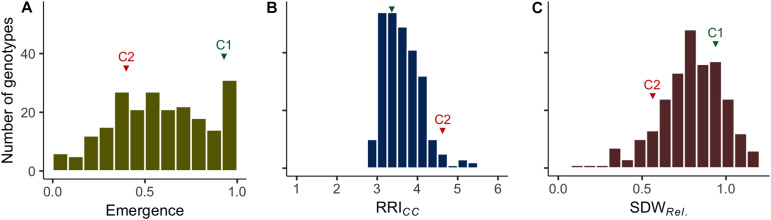
Frequency distributions of the estimated means of **(A)** plant emergence rate (Emergence), **(B)** root rot index (RRI*_*CC*_*; levels 1–6) and **(C)** relative shoot dry weight (SDW*_*Rel.*_*) assessed on 261 pea lines after 14 days (Emergence) or 21 days (RRI*_*CC*_* and SDW*_*Rel.*_*) under controlled conditions on infested soil. The means of reference cultivars C1 (tolerant) and C2 (susceptible) are indicated in green and red, respectively.

Clear pathogenesis was observed over all lines and replications 21 days after sowing, with significant lower plant emergence, plant height and shoot biomass in the infested soil compared to the sterile control (Table 1 and Supplementary Figure 1). Disease development ranged from single localized lesions on the root and the lower stem to heavily decayed root systems. Most plants showed an intermediate infection with light to dark brown discoloration and reduced volume of the root system (Supplementary Figure 2). Reproducible differentiation was achieved and the two reference cultivars fit the expected response, with C1 showing significantly higher emergence rate and relative shoot dry weight (SDW*_*Rel.*_*) and lower root rot RRI*_*CC*_* than C2 (Figure 1 and Supplementary Figure 3). High heritability was found for plant height, shoot biomass and plant emergence in both the infested soil and the sterilized soil, ranging from 0.76 to 0.96 and from 0.93 to 0.99, respectively (Table 1). In 67 out of 1,092 pots of the sterile control treatment, at least one plant showed nodule formation. No significant correlation was found between nodulation index and shoot fresh weight (*r*_*s*_ = 0.21, *p* = 0.09) or shoot dry weight (*r*_*s*_ = 0.18, *p* = 0.15). In a preliminary experiment we compared the growth of rhizobia inoculated (ProGrow-PRX 753, Progress, Germany) and non-inoculated pea plants grown on sterilized soil. No significant differences were found for biomass measurements 28 days after sowing (data not shown). Hence, data from nodulated control plants were not excluded from the analysis.

**TABLE 1:** Means, ranges, genotypic (*σ*^2^_*g*_) and residual (*σ*^2^_ε_) variance components and broad-sense heritability (*H*^2^) with standard errors (SE) for the phenotypic traits evaluated in 261 pea lines in the controlled conditions resistance screening.

Trait		Mean	Range	Variance components		*H*^2^ ± SE^*a*^
				*σ*^2^_*g*_	*σ*^2^_ε_	
**Infested soil**						
Plant height (cm)		24.0	2.0–54.7	0.314	0.059	0.96 ± 0.005
Shoot fresh weight (g)		1.0	0.1–2.9	0.020	0.025	0.76 ± 0.025
Shoot dry weight (g)		0.12	0.01–0.40	0.027	0.030	0.78 ± 0.022
Relative shoot fresh weight	SFW*_*Rel.*_*	0.65	0.06–1.81	0.008	0.040	0.46 ± 0.057
Relative shoot dry weight	SDW*_*Rel.*_*	0.82	0.03–2.14	0.014	0.053	0.51 ± 0.052
Relative root fresh weight	RDW*_*Rel.*_*	0.29	0.0002–0.94	0.005	0.014	0.58 ± 0.053
Relative root dry weight	RDW*_*Rel.*_*	0.42	0.0019–1.25	0.009	0.034	0.51 ± 0.066
Root rot index	RRI*_*CC*_*	3.7	2–6	0.067	0.355	0.43 ± 0.059
Cortex decay index	CDI	2.4	1–5	0.114	1.425	0.24 ± 0.081
Shoot lesion index	SLI	3.7	1–6	0.250	1.500	0.40 ± 0.065
Disease height (cm)	DIS	2.2	0–6	0.379	1.014	0.60 ± 0.043
Wilted nodes ratio^*b*^	WIL	0.34	−0.38 to 1	0.011	0.056	0.43 ± 0.060
Nodulation index	NOD	2.5	1–5	0.547	0.923	0.70 ± 0.031
Emergence rate		0.6	0–1	1.99	1.000	0.89 ± 0.013
**Sterilized soil**						
Plant height (cm)		29.7	6.3–64.0	0.299	0.009	0.99 ± 0.001
Shoot fresh weight (g)		1.6	0.2–3.8	0.015	0.004	0.94 ± 0.006
Shoot dry weight (g)		0.15	0.03–0.49	0.022	0.007	0.93 ± 0.007
Emergence rate		0.95	0.25–1	NA^*c*^	NA	NA

**Plants were grown in naturally infested and sterilized field soil under controlled conditions in four replications and evaluated 21 days after sowing. ^*a*^SE of variance components and H^2^ were estimated by bootstrap (1,000 iterations) using the bootMer() function in R. ^*b*^The wilted nodes ratio was corrected by subtraction of the wilted nodes ratio in the corresponding control (sterile) pot. ^*c*^The calculation of variance components with the R function lmer() is not possible on this data due to missing variation.**

### Resistance Screening of 261 Pea Landraces, Breeding Lines and Cultivars

The number of emerged plants per pot 14 days after sowing in the infested field soil differed significantly among pea lines (*p* < 0.0001; pea lines with a seed germination rate below 0.85 in the control treatment (seven lines; Supplementary Figure 1) were excluded from the analysis of emergence). Relative shoot and root biomass, i.e., the ratio between the infested and the sterilized soil treatment, were calculated to assess disease-related growth performance of the pea lines in the infested field soil (Table 1). Relative shoot fresh weight (SFW*_*Rel.*_*) and SDW*_*Rel.*_* showed significant differences (*p* < 0.0001) between lines, ranging from 0.06 to 1.81 (mean = 0.65) and from 0.03 to 2.14 (mean = 0.82), respectively. Relative root fresh (RFW*_*Rel.*_*) and dry (RDW*_*Rel.*_*) weights also showed significant differences (*p* < 0.0001) between lines, ranging from 0.0002 to 0.94 (mean = 0.29) and 0.0019 to 1.25 (mean = 0.42), respectively. Significant pea line effects were also found for RRI*_*CC*_*, cortex decay (CDI), shoot lesion (SLI), disease progress (DIS), wilted nodes ratio (WIL) and nodulation (NOD) (*p* < 0.0001). Linear model estimated means, including *p*-values for the fixed effects and model marginal *R*^2^ for all traits are given in Supplementary Table 5.

The estimate of heritability for plant emergence in the infested soil was high (*H*^2^ = 0.89, Table 1). Very high heritability values were obtained for plant height (*H*^2^ = 0.96 in the infested soil and 0.99 in the sterilized soil) as well as for fresh and dry shoot weight in sterilized soil (*H*^2^ = 0.94 and 0.93, respectively). Moderate to high heritabilities (*H*^2^ = 0.4–0.7) were found for relative biomasses, RRI*_*CC*_*, SLI, DIS, WIL, and NOD. Only CDI showed a low heritability of *H*^2^ = 0.24.

### Relation Between Disease Parameters

**FIGURE 2:**
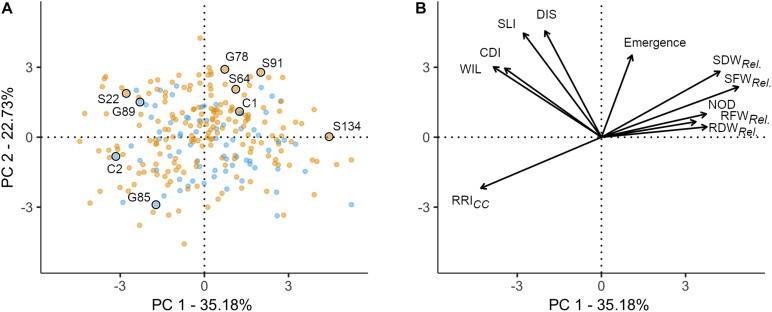
Principal component analysis of 261 pea genotypes and 11 phenotypic traits assessed under controlled conditions in infested soil. The first two principal components are shown, accounting for 57.91% of the variance in the data set. **(A)** Individuals factor map; nine pea lines evaluated in the field experiment are explicitly labeled. Blue and orange dots represent semi leafless and full leaf type genotypes, respectively. **(B)** Factor map of the 11 variables; the coordinates of the variables were multiplied by seven to produce clear visual display. Emergence = emergence rate; RFW*_*Rel.*_*, RDW*_*Rel.*_*, SFW*_*Rel.*_*, and SDW*_*Rel*_*, relative root and shoot fresh and dry weight, respectively; NOD, nodulation index; RRI, root rot index; WIL, wilted nodes; CDI, cortex decay index; SLI, shoot lesion index; DIS, disease progress.

A PCA was performed to explore the relationship among 11 traits assessed in the controlled conditions resistance screen for 261 pea lines. The two first principal components explained 57.9% of the total variance (PC1: 35.2% and PC2: 22.7%; Figure 2). The 11 traits resulted in four distinct groups: (i) relative biomass measurements and NOD (upper right quadrant); (ii) RRI*_*CC*_* (lower left quadrant); (iii) WIL, CDI, SLI, and DIS (upper left quadrant); and (iv) plant emergence between group (i) and (iii) (Figure 2B). In the first group, relative shoot fresh and dry biomass are well represented on the first axis (cos^2^ > 0.49). RRI*_*CC*_* is pointing in opposite direction and is also well represented on the first axis (cos^2^ = 0.51). On the second axis, emergence (cos^2^ = 0.34), SLI (cos^2^ = 0.55) and DIS (cos^2^ = 0.58) are well represented. CDI and WIL are equally well represented on axis 1 and 2. Pea lines with extreme positive (upper right) or negative coordinates (lower left) were considered as the most resistant or susceptible lines, respectively (Figure 2A). Generally, the dispersion of the pea lines in the two first dimensions showed that the frequency of resistant and susceptible lines was homogeneous among the evaluated collection. The position of the two reference cultivars are according to the expectations, with C1 emerging well and being more resistant (lower RRI*_*CC*_* and higher relative shoot weight) and C2 poorly emerging and being highly susceptible (high RRI*_*CC*_* and low relative shoot weight). No grouping of the evaluated pea lines according to leaf type was detected with respect to the eleven traits assessed.

**FIGURE 3:**
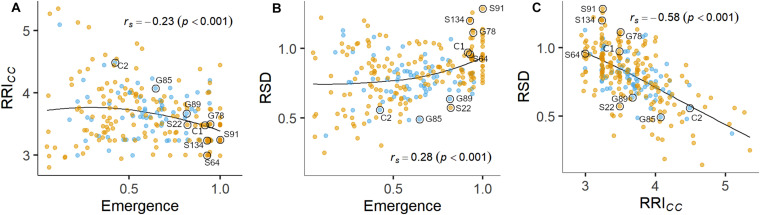
Pairwise relations between linear-mixed model estimated means of **(A)** root rot index (RRI*_*CC*_*) and plant emergence rate, **(B)** relative shoot dry weight (SDW*_*Rel.*_*) and plant emergence rate and **(C)** SDW*_*Rel.*_* and RRI*_*CC*_* assessed on 261 pea lines after 14 days (Emergence) or 21 days (RRI*_*CC*_* and SDW*_*Rel.*_*) under controlled conditions in infested soil. Rank correlation coefficients (Spearman’s *rho*) and associated *p*-values are displayed in the plots. A LOESS regression line is included in each panel. Nine pea lines evaluated in the field experiment are labeled. Blue and orange dots represent semi leafless and full leaf type genotypes, respectively.

Plant emergence rate, RRI*_*CC*_* and SDW*_*Rel.*_* were selected for further examination (Figures 1, 3, and Supplementary Figure 4). Genotypic means of emergence and SDW*_*Rel.*_* showed considerable variation among the evaluated 261 pea lines. Emergence rate in the infested soil showed a bimodal distribution, with 22% of the pea lines having an emergence rate ≥ 0.9 in the infested soil. In contrast, RRI*_*CC*_* showed less variation and a strong truncated distribution with a positive skew toward susceptibility. Most pea lines got an average score between 3 and 4 and no line got the score 1 (healthy, no symptoms) or 2 (single, localized lesions). Relative shoot dry weight (SDW*_*Rel.*_*) showed a close to normal distribution with negative skew toward susceptibility and good differentiation between the pea lines. All three variables were significantly correlated with each other (Figure 3). Emergence showed a weak negative rank correlation with RRI*_*CC*_* (*r*_*s*_ = − 0.23, *p* < 0.0001) and a weak positive correlation with SDW*_*Rel*__._* (*r*_*s*_ = 0.28, *p* < 0.0001). SDW*_*Rel.*_* showed a medium negative correlation with RRI*_*CC*_* (*r*_*s*_ = − 0.58, *p* < 0.0001).

Relative root and relative shoot biomass were moderately correlated with each other (*r_*s*_* = 0.32–0.44, *p* < 0.001). Shoot lesion index was positively correlated with cortex decay (*r*_*s*_ = 0.61, *p* < 0.001), disease progress in stem base (*r*_*s*_ = 0.67, *p* < 0.001) and wilted nodes ratio (*r*_*s*_ = 0.55, *p* < 0.001). NOD was positively correlated with SFW*_*Rel*__._* (*r*_*s*_ = 0.50, *p* < 0.001) and negatively correlated with RRI*_*CC*_* (*r*_*s*_ = −0.58, *p* < 0.001) (Supplementary Figure 4).

### On Farm Verification of the Controlled Conditions Resistance Screening

**FIGURE 4:**
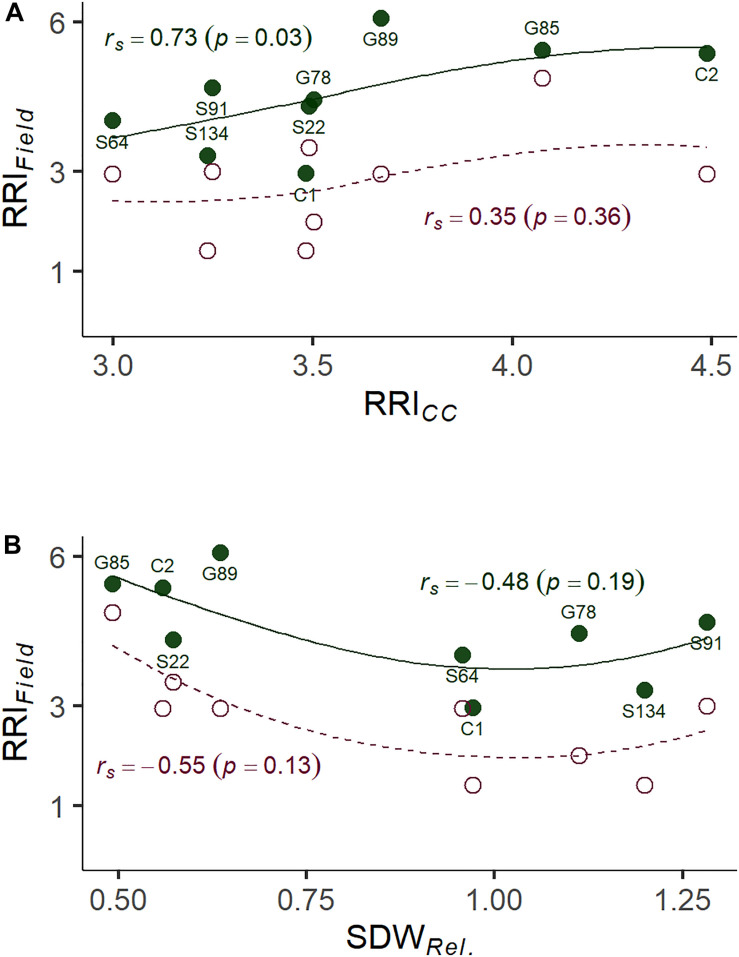
**(A)** Correlation between root rot assessed under controlled conditions on infested soil 21 days after sowing (RRI*_*CC*_*; 1: no symptoms—6: plant dead) and root rot assessed in the on-farm experiment (RRI*_*Field*_*; 1: no symptoms—8: plant dead). **(B)** Correlation between relative shoot dry weight assessed under controlled conditions (SDW*_*Rel.*_*) and RRI*_*Field*_* of nine pea lines. The nine pea lines with contrasting resistance phenotypes were evaluated on a field site with heavy pea root rot complex (PRRC) infestation (closed dots, solid LOESS line), and on a field site with moderate PRRC infestation (open dots, dashed LOESS line). Estimated means are presented for the nine field evaluated lines. Rank correlation coefficients (Spearman’s *rho*) and associated *p*-values are indicated for both field sites.

A subset of nine pea lines, including the two reference cultivars, was evaluated for PRRC resistance in the field in order to verify the results of the resistance screen under controlled conditions (Table 2). Significant genotype (*p* = 0.009) and field site effects (*p* = 0.0001) were found for RRI*_*Field*_*. The genotype x field site interaction was not significant (*p* = 0.49). Genotypic means ± SE of root rot index ranged from 3.0 ± 0.6 to 6.0 ± 0.0 in the heavily infested field site. Root rot was lower in the adjacent field site with moderate root rot potential, ranging from 1.5 ± 0.5 to 4.5 ± 1.5. The two check cultivars fit the expected response, with C1 showing lower RRI*_*Field*_* than C2 in both field sites. A significant rank correlation was found between RRI*_*CC*_* and RRI*_*Field*_* in the heavily infested field site (*r_*s*_* = −0.73, *p* = 0.03; Figure 4A). No significant correlation was found between RRI*_*CC*_* and RRI*_*Field*_* in the moderately infested field site or between RRI*_*Field*_* and SDW*_*Rel*__._* (Figure 4).

**TABLE 2:** Means of relative shoot dry weight (SDW*_*Rel.*_*), root rot index (RRI*_*CC*_* and RRI*_*Field*_*) and emergence rate for nine pea genotypes evaluated under controlled conditions and in on-farm field trails.

	Controlled conditions			Field sites	
				Heavy infestation	Moderate infestation
Genotype	Emergence	RRI*_*CC*_*	SDW*_*Rel.*_*	RRI*_*Field*_*	RRI*_*Field*_*
**Reference cultivars**					
C1	0.92 [0.86, 0.95]	3.48 [3.3, 3.67]	0.97 [0.89, 1.04]	2.97 [1.52, 4.4]	1.42 [−0.68, 2.85]
C2	0.42 [0.34, 0.51]	4.43 [4.21, 4.64]	0.57 [0.48, 0.65]	5.38 [3.77, 7.41]	2.95 [1.18, 4.71]
**Field tested lines**					
S91	1 [0, 1]	3.24 [2.65, 3.83]	1.23 [1, 1.45]	4.69 [3.28, 6.17]	2.99 [1.23, 4.76]
S134	0.93 [0.62, 0.99]	3.23 [2.64, 3.82]	1.16 [0.9, 1.43]	3.32 [1.88, 4.77]	1.42 [−0.68, 2.85]
G78	0.95 [0.69, 0.99]	3.50 [2.91, 4.09]	1.09 [0.87, 1.32]	4.45 [2.66, 6.28]	2.00 [0.12, 3.66]
S64	0.93 [0.62, 0.99]	2.98 [2.39, 3.56]	0.96 [0.73, 1.18]	4.03 [2.58, 5.47]	2.95 [1.18, 4.71]
G89	0.82 [0.5, 0.94]	3.67 [3.08, 4.26]	0.64 [0.41, 0.86]	6.08 [5.59, 9.22]	2.95 [1.18, 4.71]
S22	0.82 [0.56, 0.94]	3.49 [2.9, 4.08]	0.58 [0.35, 0.81]	4.33 [2.52, 6.15]	3.49 [1.72, 5.26]
G85	0.65 [0.39, 0.84]	4.07 [3.48, 4.65]	0.51 [0.28, 0.73]	5.45 [4.25, 7.14]	4.88 [3.18, 6.71]

**Estimated means, followed by the upper and lower 95% confidence interval limits are presented for controlled conditions and field data. Lines are sorted by SDW_*Rel.*_ in descending order.**

### Fungal Community in Pea Roots Grown in Infested Soil Under Controlled Conditions

Sequencing of the ITS1 region from total DNA extracted from bulk soil, rhizosphere soil and root revealed a total of 1,190,412 high-quality sequences with a median of 55,670 sequences per sample. The rarefaction analysis showed that samples reached an asymptote, maximizing the number of distinguishable operational taxonomic units (OTUs), with decreasing OTU richness from bulk soil to rhizosphere soil to root samples (Supplementary Figure 5). There was no significant differentiation between the two pea lines; therefore, the sequencing data was pooled for further analysis. Among the most abundant OTUs present in pea roots, sequences could be assigned to several putative pea pathogens including several *Fusarium* spp., *Rhizoctonia solani*, *Didymella* sp., and other putative plant pathogens (Table 3 and Supplementary Table 4). Putative plant beneficial fungi included *Clonostachys rosea* (5th most abundant taxa in roots), *Coprinellus* sp. and several members of arbuscular mycorrhizal fungi (AMF), e.g., *Funneliformis* spp., *Entrophspora* sp., and *Diversispora* spp.

**TABLE 3:** Taxonomic information and mean relative abundance of the 20 most abundant operational taxonomic units (OTUs) and further selected OTUs in bulk soil (*n* = 4), rhizosphere soil (*n* = 8), and root (*n* = 8) of 21 days old pea grown in infested soil under controlled conditions.

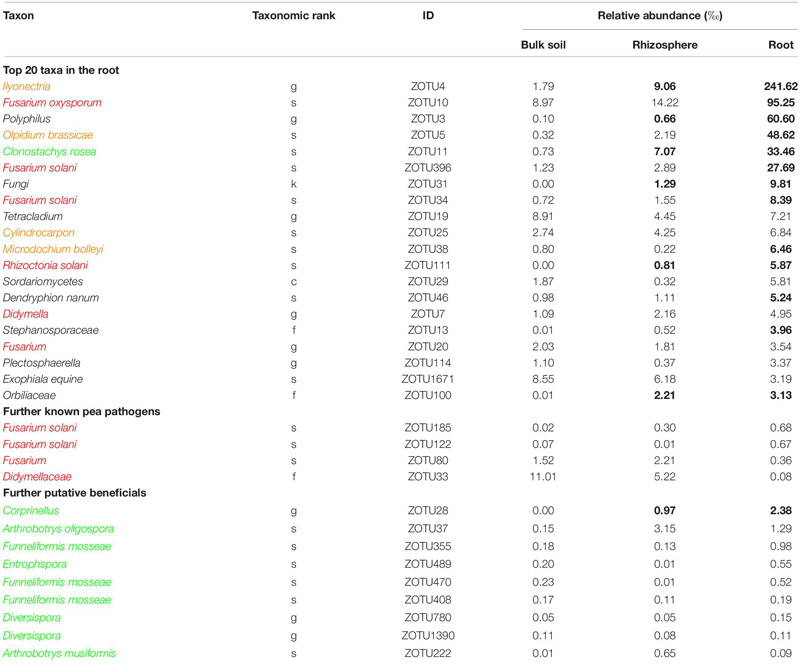

**Bold values highlight significantly enriched OTUs compared with bulk soil (FDR-adjusted p < 0.05).^*a*^red = putative pea pathogen, orange = putative plant pathogen, blue = putative plant beneficial.^*b*^s, species; g, genus; f, family; c, class; k, kingdom.**

## Discussion

There is increasing evidence that plants should be recognized as a holobiont with their health status largely depending on well-balanced networks within their microbial community (Berg et al., 2017). It has been shown that the plant genotype influences the microbiome composition and that the microbial community can be shaped by plant breeding (Horton et al., 2014; Pérez-Jaramillo et al., 2017). From this point of view, we designed a resistance screening where 261 pea landraces, breeding lines and cultivars were grown under controlled conditions in naturally infested field soil harboring a complex of pea root rot pathogens and non-pathogenic, potentially beneficial microbes.

### Evaluation of the PRRC Resistance Screening

Plant emergence in the infested soil showed ample genotypic variation and high heritability. Poor emergence and damping-off is a constant threat to pea production, especially in the temperate regions, where peas are spring-sown under cool and wet conditions. Damping-off can be a major yield limiting factor and cause severe economic loss (Lamichhane et al., 2017). Screening for resistance against damping-off based on artificial inoculation was shown to be poorly correlated with resistance scorings obtained in the field (Muehlbauer and Kraft, 1973). By contrast, we show that plant emergence can be easily and reproducibly assessed in a complex system and represents a valuable trait for assessing resistance of pea lines against damping-off and early stages of root rot.

The root rot index assessed under controlled conditions (RRI*_*CC*_*) allowed to differentiate between highly susceptible and partially resistant pea lines. The heritability of RRI*_*CC*_* was found to be similar to the results of previous studies on root rot pathogens in pea. Desgroux et al. (2016) reported heritabilities as high as 0.9 for artificial inoculations with single isolates of *A. euteiches*, however heritability was as low as 0.28 in their field experiments. Muehlbauer and Kraft (1973) screened pea lines for resistance on field soil heavily infested with *F. solani* and *P. ultimum* and found a heritability of 0.44, similar to our experiment. Thus, the assessment of a root rot index is useful for breeding nurseries and field trials as it allows to quantify complex disease expressions and provides a direct estimation of resistance capacities.

The assessment of relative biomass allowed to compare biomass loss among morphologically highly differentiated pea lines of different leaf type and agronomic end-use. Relative biomass measurements showed abundant variation and allowed to differentiate between highly susceptible and partially resistant pea lines. We found similar pea shoot biomass reductions and associated heritabilities as previously reported from artificial inoculation assays with isolates of *Fusarium avenaceum*, *F. oxysporum*, and *F. solani* (Šišić et al., 2018) and *Aphanomyces euteiches* (Pilet-Nayel et al., 2005). However, measuring relative biomass requires that each genotype is tested twice on sterilized and on infested soil, requiring more resources. By sterilization, possible plant-microbe interactions, including plant beneficial interactions, are eliminated in the control treatment. The employment of sterilized soil as a control is therefore a compromise allowing to compare growth of a morphologically diverse collection of pea lines. Comparing the growth performance of pea lines in infested field sites to non-infested field sites stands for a practical alternative to sterile soil control. This would further benefit from higher biological significance, as the non-infested soil harbors a resident microbial community. However, multi-site experiments need to be planned carefully, as possible interactions between the plant genotype, different properties of the soil (e.g., physico-chemical or microbial), agricultural management and climatic conditions can significantly influence the results and the conclusions drawn upon them (Busby et al., 2017).

Relative biomass measurements represent valuable surrogate traits for plant health and their relation to other disease symptoms of the plant allows for further exploration of genotypic differences. Several pea lines showed below-average RRI*_*CC*_* and above-average SDW*_*Rel.*_*, indicating exploitable enhanced disease resistance. None of the evaluated lines showed full root rot resistance, but considerable variation in SDW*_*Rel.*_* was evidenced for lines with RRI*_*CC*_* values between 3 and 4. Few pea lines even showed high RRI*_*CC*_* while still growing above average, indicating enhanced levels of disease tolerance for these lines, where tolerance is the ability of plants to perform well when infected with a pathogen. Conner et al. (2013) found significant differences in tolerance against Aphanomyces root rot infection in a collection of pea breeding lines in the field. They concluded that above-ground plant growth traits could be useful in the selection of breeding lines with tolerance to Aphanomyces root rot, still producing high yield despite the infection. Based on the proposed screening system, we suggest to select lines with high relative biomass and low RRI*_*CC*_* for further field assessments and as possible resistance sources against the root rot complex, as breeders will ultimately be interested in both, disease resistance (low levels of disease symptoms) and disease tolerance (low yield depression when symptoms occur in the target environment).

Estimates of heritability for disease-related traits calculated from single-isolate inoculations and controlled conditions screenings are typically higher compared with field experiments or infested soil-based systems because random experimental variance can be minimized. Single-isolate experiments are highly valuable for linkage-mapping and genome-wide association studies (GWAS). Various published studies employed artificial inoculation resistance screenings and successfully identified loci controlling partial resistance against individual root rot pathogens of pea (Hamon et al., 2013; Pilet-Nayel et al., 2017; Desgroux et al., 2018; Coyne et al., 2019). However, they assume that the different pathogens have additive effects and ignore the complex interactions between pathogenic and other microorganisms under farming conditions.

Here we show that considerable heritability for resistance traits can be found in systems based on infested field soil. The simple and resource efficient setup makes the screening system a valuable tool for early resistance breeding efforts. No prior knowledge on the presence and composition of specific pathogens in the soil is needed. Furthermore, selection rounds independent of the field season are possible with the screening system. In our on-farm experiment, we were able to confirm two distinct infestation levels in two close-by field sites and found a significant genotype effect for RRI*_*Field*_*. Our field results confirm the applicability of the controlled conditions screening system to predict adult plant field resistance, with a significant correlation between RRI*_*CC*_* and RRI*_*Field*_* in the highly infested site. On the other hand, in the close-by moderately infested site, disease development was low and no significant correlation with the controlled conditions screening could be found. Because of the proximity of the two sites we can rule out substantial environmental differences with respect to physical soil parameters and climatic conditions. We assume differences in the microbial composition in the soil to be responsible for the distinct pathogenicity levels. Our results suggest that the predictability of the field performance depends on the disease pressure in the field. Thus, the developed screening assay should be used to screen genotypes in different naturally infested soil types to predict possible GxE interactions accounting for different compositions of relevant pathogens or other microbial species.

The experimental design of this soil-based screening system requires replication and, thus, cannot be used for single plant selection in early generations but only for advanced generations when sufficient homogeneity is given (e.g., F_4_, F_5_ lines). For selection in earlier generations molecular tools need to be developed. The moderate to high heritability values obtained for the different resistance traits under controlled conditions would allow for GWAS or genomic prediction. These molecular resources would not only allow to identify possible PRRC resistance loci but also to disentangle innate and microbiome-mediated resistance mechanisms when paired with root microbiome data. Then, these genomic tools would represent a powerful tool to study the role of the plant-associated microbial community in PRRC resistance.

### Plant Resistance in the Light of Pathogen Complexes

The ITS-amplicon sequencing allowed to detect several pathogenic fungi in the soil and root of diseased peas. Taxa including known members of pea pathogens *F. oxysporum*, *F. solani*, and *R. solani* were enriched in the roots compared to the bulk soil. These three pathogens belong to the most important root rot pathogens of pea in the temperate zones (Mc Phee et al., 2012; Chittem et al., 2015; Coyne et al., 2015; Melzer et al., 2016). *Fusarium solani* and various isolates of *Rhizoctonia solani* are also causal agents of damping-off in pea (Melzer et al., 2016; Lamichhane et al., 2017). The high abundance of these pathogens in diseased roots indicates their probable role in the reduction of plant emergence and later disease expression. The sequencing data revealed the presence of two taxa, *Didymellaceae* and *Didymella* sp. which indicates the role of any of the two major pea pathogens *Didymella pinodella* and *Didymella pinodes* in PRRC in the studied soil. Both pathogens have been frequently isolated from diseased peas in German fields (Pflughöft et al., 2012; Baćanović-Šišić et al., 2018). Furthermore, the two taxa *Ilyonectria* and *Olpidium brassicae* were enriched in the roots. Both taxa are plant pathogens with a wide host range and have been detected in earlier microbiome studies of field pea (Yu et al., 2012a,b). However, their status in relation to pea root rot remains elusive, as they are not known pea pathogens. Lay et al. (2018) stated that the unresolved taxonomy of the *Olpidium* complex could lead to an overrepresentation in amplicon sequencing studies. The demonstrated co-occurrence of pea root rot pathogens emphasizes the PRRC concept, as already stated in other studies (Chittem et al., 2015; Taheri et al., 2017; Willsey et al., 2018).

Co-inoculation of fungal and oomycotan pathogens (e.g., *P. ultimum*—*F. oxysporum* or *R. solani*—*F. solani*—*A. euteiches* in pea and *P. ultimum*—*A. euteiches* in bean) have been show to significantly increase disease development (Kerr, 1963; Pfender and Hagedorn, 1982; Shehata et al., 1983). The utilized primer pair specifically targets members of the kingdom Fungus, underrepresenting oomycetes. Therefore, our sequencing data does not allow for clear statements about the presence of *Aphanomyces euteiches*, *Pythium* sp. or *Phytophtora* sp. These oomycetes are also important pea pathogens causing damping-off and root rot (Gaulin et al., 2007; Heyman et al., 2013; Alcala et al., 2016). Symptoms of seedlings showing damping-off included water-soaked, decaying seeds and rotting of the hypocotyl, possible indications of the presence of *Pythium* spp.; at later stages roots were honey-brown and water-soaked, indicating *A. euteiches* to be involved in the PRRC. Additional molecular analyses with species-specific primers are required to analyze these oomycotan pathogens.

In addition to the pathogenic taxa, the ITS-amplicon sequencing allowed to detect plant beneficial fungal taxa. The taxa *Clonostachys rosea* and *Corprinellus* were significantly enriched in the roots. *Clonostachys rosea* is able to colonize seeds and young pea plantlets and is a known antagonist of fungal pathogens. It has been shown to significantly limit the growth of major PRRC pathogens *in vitro* and to reduce disease in the field (Xue, 2003a,b). Members of the taxon *Corprinellus* produce anti-bacterial and anti-fungal compounds (Zahid et al., 2006) and an isolate of *C. curtus* was shown to reduce growth of *Rhizoctonia solani* in the rhizosphere of cabbage (Nakasaki et al., 2007). Various sequences were also attributed to three taxa belonging to AMF, well-known plant symbionts gaining recent attention for AMF-mediated disease resistance (Hohmann and Messmer, 2017). Bioprotection of pea by AMF against the pathogen *A. euteiches* has been repeatedly reported (Bodker et al., 1998; Slezack et al., 2000; Li et al., 2019). In contrast to the before-mentioned direct fungal antagonists, AMF’s action is indirect: they induce plant resistance and regulate the plant defense mechanisms (Jung et al., 2012).

Our sequencing data revealed a diverse collection of fungal pathogens and putative beneficials present in the roots of infected pea plants. This finding confirms the study of Xu et al. (2012), where the health status of pea (infected or healthy) only affected the fungal community composition in the roots, but not in rhizosphere or bulk soil. The data suggests that the analysis of bulk soil alone does not allow to assess the occurrence of specific pathogens because pea pathogens and fungal antagonists were specifically enriched in the roots, in comparison to the bulk soil. Future research is necessary to compare groups of resistant and susceptible plant genotypes in different infested soils in order to validate causal agents of PRRC and identify diversity indices or key taxa involved in microbiome-mediated disease resistance. In this regard, it will be particularly critical to explore if different pea lines that show comparable disease resistance harbor similar or different levels of key microbes and to determine whether resistant lines respond differently to key pathogens. In addition to understanding the interaction of the major PRRC pathogens with the plant and their importance in determining disease susceptibility or resistance future research should also consider the whole microbial diversity of PRRC pathosystems. Research in this field will contribute in further disentangling the contribution of the plant genotype, the abiotic soil environment and the root associated microbiome to plant health, as proposed recently by Oyserman et al. (2019). Eventually, this could lead to the development of molecular markers, either on the microbiome or the plant side, that allow the prediction of plant resistance under complex field conditions and the selection of breeding material.

## Conclusion

This study demonstrates the value of controlled conditions resistance screenings in predicting the performance of pea lines in a high PRRC-infested field site. The resistance screening assay reproducibly identified partially resistant and highly tolerant pea lines despite the complexity of the fungal community in the used substrate. The simple technical setup and ease of applicability in comparison to field trials make the screening system suitable for early selection in resistance breeding programmes. Heritabilities of the assessed resistance traits show promise to use the screening system in molecular and conventional pea breeding, and therefore to strengthen resistance breeding of this ecologically and economically invaluable crop. The use of agricultural soils allows to screen for plant resistance mechanisms of the entire ecological unit consisting of the plant and its associated microbial community. This is assumed to be one of the main reasons for the strong correlation between controlled and field conditions performance. For future lines of research, it will be revealing to link plant performance, host genetics and microbiome diversity and functions to assess plant health at the holobiont level. This holistic approach will broadly support breeding of pea and other major crops and promote sustainable food production.

## Data Availability Statement

The amplicon sequencing datasets generated for this study can be found in the European Nucleotide Archive under the accession number PRJEB39076.

## Author Contributions

PH, MM, and BS conceived the study. LW, MM, and PH designed the experiments. LW carried out the experimental work of the resistance screening and the field trial, analyzed the data, and wrote the manuscript. PH and NB conducted experimental work and data analysis of the microbiome study. NB, PH, MM, and BS contributed to the writing of the manuscript. All authors contributed to the interpretation of results, read and approved the final manuscript.

## Conflict of Interest

The authors declare that the research was conducted in the absence of any commercial or financial relationships that could be construed as a potential conflict of interest.

## Footnotes

1 https://www.ars-grin.gov

2 https://www.gzpk.ch/

3 https://openwetware.org/wiki/SPRI_bead_mix

## References

[B1] Abarenkov K. Nilsson R. H. Larsson K. H. Alexander I. J. Eberhardt U. Erland S. (2010). The UNITE database for molecular identification of fungi - recent updates and future perspectives. *New Phytol.* 186 281–285. 10.1111/j.1469-8137.2009.03160.x 20409185

[B2] Abdullah A. S. Moffat C. S. Lopez-Ruiz F. J. Gibberd M. R. Hamblin J. Zerihun A. (2017). Host–multi-pathogen warfare: pathogen interactions in co-infected plants. *Front. Plant Sci.* 8:1806. 10.3389/fpls.2017.01806 29118773 PMC5660990

[B3] Alabouvette C. Olivain C. Migheli Q. Steinberg C. (2009). Microbiological control of soil-borne phytopathogenic fungi with special emphasis on wilt-inducing Fusarium oxysporum. *New Phytol.* 184 529–544. 10.1111/j.1469-8137.2009.03014.x 19761494

[B4] Alcala A. V. C. Paulitz T. C. Schroeder K. L. Porter L. D. Derie M. L. du Toit L. J. (2016). Pythium species associated with damping-off of pea in certified organic fields in the columbia basin of central Washington. *Plant Dis.* 100 916–925. 10.1094/PDIS-07-15-0774-RE 30686151

[B5] Baćanović-Šišić J. Šišić A. Schmidt J. H. Finckh M. R. (2018). Identification and characterization of pathogens associated with root rot of winter peas grown under organic management in Germany. *Eur. J. Plant Pathol.* 151 745–755. 10.1007/s10658-017-1409-0

[B6] Bainard L. D. Navarro-Borrell A. Hamel C. Braun K. Hanson K. Gan Y. (2017). Increasing the frequency of pulses in crop rotations reduces soil fungal diversity and increases the proportion of fungal pathotrophs in a semiarid agroecosystem. *Agric. Ecosyst. Environ.* 240 206–214. 10.1016/j.agee.2017.02.020

[B7] Bates D. Maechler M. Bolker B. Walker S. (2015). Fitting linear mixed-effects models ssing lme4. *J. Stat. Softw.* 67 1–48. 10.18637/jss.v067.i01

[B8] Berg G. Köberl M. Rybakova D. Müller H. Grosch R. Smalla K. (2017). Plant microbial diversity is suggested as the key to future biocontrol and health trends. *FEMS Microbiol. Ecol.* 93 1–9. 10.1093/femsec/fix050 28430944

[B9] Bodah E. T. Porter L. D. Chaves B. Dhingra A. (2016). Evaluation of pea accessions and commercial cultivars for fusarium root rot resistance. *Euphytica* 208 63–72. 10.1007/s10681-015-1545-6

[B10] Bodenhausen N. Somerville V. Desirò A. Walser J.-C. Borghi L. van der Heijden M. G. A. (2019). Petunia- and arabidopsis-specific root microbiota responses to phosphate supplementation. *Phytobiomes J.* 3 112–124. 10.1094/pbiomes-12-18-0057-r

[B11] Bodker L. Kjoller R. Rosendahl S. (1998). Effect of phosphate and the arbuscular mycorrhizal fungus Glomus intraradices on disease severity of root rot of peas (*Pisum sativum*) caused by *Aphanomyces euteiches*. *Mycorrhiza* 8 169–174. 10.1007/s005720050230

[B12] Busby P. E. Soman C. Wagner M. R. Friesen M. L. Kremer J. Bennett A. (2017). Research priorities for harnessing plant microbiomes in sustainable agriculture. *PLoS Biol.* 15:e2001793. 10.1371/journal.pbio.2001793 28350798 PMC5370116

[B13] Chittem K. Mathew F. M. Gregoire M. Lamppa R. S. Chang Y. W. Markell S. G. (2015). Identification and characterization of *Fusarium* spp. associated with root rots of field pea in North Dakota. *Eur. J. Plant Pathol.* 143 641–649. 10.1007/s10658-015-0714-8

[B14] Conner R. L. Chang K. F. Hwang S. F. Warkentin T. D. Mcrae K. B. (2013). Assessment of tolerance for reducing yield losses in field pea caused by Aphanomyces root rot. *Can. J. Plant Sci.* 93 473–482. 10.4141/Cjps2012-183

[B15] Coyne C. J. Pilet-Nayel M.-L. L. McGee R. J. Porter L. D. Smýkal P. Grünwald N. J. (2015). Identification of QTL controlling high levels of partial resistance to *Fusarium solani* f. sp. *pisi* in pea. *Plant Breed.* 134 446–453. 10.1111/pbr.12287

[B16] Coyne C. J. Porter L. D. Boutet G. Ma Y. McGee R. J. Lesne A. (2019). Confirmation of Fusarium root rot resistance QTL Fsp-Ps 2.1 of pea under controlled conditions. *BMC Plant Biol.* 19:98. 10.1186/s12870-019-1699-9 30866817 PMC6417171

[B17] Desgroux A. Anthoëne V. L. Roux-Duparque M. Rivière J.-P. Aubert G. Tayeh N. (2016). Genome-wide association mapping of partial resistance to *Aphanomyces euteiches* in pea. *BMC Genomics* 17:124. 10.1186/s12864-016-2429-4 26897486 PMC4761183

[B18] Desgroux A. Baudais V. N. Aubert V. Le Roy G. de Larambergue H. Miteul H. (2018). Comparative genome-wide-association mapping identifies common loci controlling root system architecture and resistance to *Aphanomyces euteiches* in pea. *Front. Plant Sci.* 8:2195. 10.3389/fpls.2017.02195 29354146 PMC5761208

[B19] Duc G. Agrama H. Bao S. Berger J. Bourion V. De Ron A. M. (2015). Breeding annual grain legumes for sustainable agriculture: new methods to approach complex traits and target new cultivar ideotypes. *Crit. Rev. Plant Sci.* 34 381–411. 10.1080/07352689.2014.898469

[B20] Edgar R. C. (2010). Search and clustering orders of magnitude faster than BLAST. *Bioinformatics* 26 2460–2461. 10.1093/bioinformatics/btq461 20709691

[B21] Edgar R. C. (2016). SINTAX: a simple non-Bayesian taxonomy classifier for 16S and ITS sequences. *bioRxiv* [Preprint]. 10.1101/074161

[B22] FAO, (2019). *FAOSTAT.* Rome: FAO.

[B23] Foyer C. H. Lam H.-M. Nguyen H. T. Siddique K. H. M. Varshney R. K. Colmer T. D. (2016). Neglecting legumes has compromised human health and sustainable food production. *Nat. Plants* 2:16112. 10.1038/NPLANTS.2016.112 28221372

[B24] Fuchs J. G. Thuerig B. Brandhuber R. Bruns C. Finckh M. R. Fliessbach A. (2014). Evaluation of the causes of legume yield depression syndrome using an improved diagnostic tool. *Appl. Soil Ecol.* 79 26–36. 10.1016/j.apsoil.2014.02.013

[B25] Gardes M. Bruns T. D. (1993). ITS primers with enhanced specificity for basidiomycetes - application to the identification of mycorrhizae and rusts. *Mol. Ecol.* 2 113–118. 10.1111/j.1365-294X.1993.tb00005.x 8180733

[B26] Gaulin E. Jacquet C. Bottin A. Dumas B. (2007). Root rot disease of legumes caused by *Aphanomyces euteiches*. *Mol. Plant Pathol.* 8 539–548. 10.1111/j.1364-3703.2007.00413.x 20507520

[B27] Goerg G. M. (2015). The lambert way to gaussianize heavy-tailed data with the inverse of Tukey’s h transformation as a special case. *Sci. World J.* 2015:909231. 10.1155/2015/909231 26380372 PMC4562338

[B28] Grünwald N. J. Coffman V. A. Kraft J. M. (2003). Sources of partial resistance to Fusarium root rot in the Pisum core collection. *Plant Dis.* 87 1197–1200. 30812722 10.1094/PDIS.2003.87.10.1197

[B29] Hamon C. Baranger A. Coyne C. J. McGee R. J. Le Goff I. L’Anthoëne V. (2011). New consistent QTL in pea associated with partial resistance to *Aphanomyces euteiches* in multiple French and American environments. *Theor. Appl. Genet.* 123 261–281. 10.1007/s00122-011-1582-z 21479935

[B30] Hamon C. Coyne C. J. McGee R. J. Lesné A. Esnault R. Mangin P. (2013). QTL meta-analysis provides a comprehensive view of loci controlling partial resistance to *Aphanomyces euteiches* in four sources of resistance in pea. *BMC Plant Biol.* 13:45. 10.1186/1471-2229-13-45 23497245 PMC3680057

[B31] Heyman F. Blair J. E. Persson L. Wikstrom M. (2013). Root rot of pea and faba bean in southern Sweden caused by Phytophthora pisi sp. *nov*. *Plant Dis.* 97 461–471. 10.1094/PDIS-09-12-0823-RE 30722231

[B32] Hohmann P. Messmer M. M. (2017). Breeding for mycorrhizal symbiosis: focus on disease resistance. *Euphytica* 213:113 10.1007/s10681-017-1900-x

[B33] Horton M. W. Bodenhausen N. Beilsmith K. Meng D. Muegge B. D. Subramanian S. (2014). Genome-wide association. *Nat. Commun.* 5:5320. 10.1038/ncomms6320 25382143 PMC4232226

[B34] Infantino A. Kharrat M. Riccioni L. Coyne C. J. McPhee K. E. Grünwald N. J. (2006). Screening techniques and sources of resistance to root diseases in cool season food legumes. *Euphytica* 147 201–221. 10.1007/s10681-006-6963-z

[B35] Jung S. C. Martinez-Medina A. Lopez-Raez J. A. Pozo M. J. (2012). Mycorrhiza-induced resistance and priming of plant defenses. *J. Chem. Ecol.* 38 651–664. 10.1007/s10886-012-0134-6 22623151

[B36] Kerr A. (1963). The root rot-Fusarium wilt complex of peas. *Aust. J. Biol. Sci.* 16 55–69. 10.1071/bi9630055

[B37] Lamichhane J. R. Dürr C. Schwanck A. A. Robin M.-H. Sarthou J.-P. Cellier V. (2017). Integrated management of damping-off diseases. A review. *Agron. Sustain. Dev.* 37 1–25. 10.1007/s13593-017-0417-y

[B38] Lay C. Y. Hamel C. St-Arnaud M. (2018). Taxonomy and pathogenicity of Olpidium brassicae and its allied species. *Fungal Biol.* 122 837–846. 10.1016/j.funbio.2018.04.012 30115317

[B39] Le S. Josse J. Husson F. (2008). FactoMineR: an R package for multivariate analysis. *J. Stat. Softw.* 25 1–18. 10.18637/jss.v025.i01

[B40] Lenth R. (2019). *emmeans**: Estimated Marginal Means, aka Least-Squares Means. R package version 1.3.3.* Available at: https://CRAN.R-project.org/package=emmeans (accessed March 19, 2019).

[B41] Li Y. Duan T. Nan Z. Li Y. (2019). Arbuscular mycorrhizal fungus alleviates alfalfa leaf spots caused by *Phoma medicaginis* revealed by RNA-seq analysis. *J Appl Microbiol.* 10.1111/jam.14387 [Epub ahead of print]. 31310670

[B42] Lundberg D. S. Yourstone S. Mieczkowski P. Jones C. D. Dangl J. L. (2013). Practical innovations for high-throughput amplicon sequencing. *Nat. Methods* 10 999–1002. 10.1038/nmeth.2634 23995388

[B43] Magoc T. Salzberg S. L. (2011). FLASH: fast length adjustment of short reads to improve genome assemblies. *Bioinformatics* 27 2957–2963. 10.1093/bioinformatics/btr507 21903629 PMC3198573

[B44] Martin M. (2011). Cutadapt removes adapter sequences from high-throughput sequencing reads. *EMBnet.journal* 17:3 10.14806/ej.17.1.200

[B45] Mc Phee K. E. Inglis D. A. Gundersen B. Coyne C. J. (2012). Mapping QTL for Fusarium wilt race 2 partial resistance in pea (*Pisum sativum*). *Plant Breed.* 131 300–306. 10.1111/j.1439-0523.2011.01938.x

[B46] Melzer M. S. Yu H. Labun T. Dickson A. Boland G. J. (2016). Characterization and pathogenicity of *Rhizoctonia* spp. from field crops in Canada. *Can. J. Plant Pathol.* 38 367–374. 10.1080/07060661.2016.1199596

[B47] Moussart A. Even M. N. Tivoli B. (2008). Reaction of genotypes from several species of grain and forage legumes to infection with a French pea isolate of the oomycete *Aphanomyces euteiches*. *Eur. J. Plant Pathol.* 122 321–333. 10.1007/s10658-008-9297-y

[B48] Muehlbauer F. J. Kraft J. M. (1973). Evidence of heritable resistance to *Fusarium solani* f. sp. *pisi* and *Pythium ultimum* in peas. *Crop Sci.* 3 34–36. 10.2135/cropsci1973.0011183X001300010011x

[B49] Müller D. B. Vogel C. Bai Y. Vorholt J. A. (2016). The plant microbiota: systems-level insights and perspectives. *Annu. Rev. Genet.* 50 211–234. 10.1146/annurev-genet-120215-034952 27648643

[B50] Nakagawa S. Schielzeth H. O’Hara R. B. (2013). A general and simple method for obtainingR2from generalized linear mixed-effects models. *Methods Ecol. Evol.* 4 133–142. 10.1111/j.2041-210x.2012.00261.x

[B51] Nakasaki K. Saito M. Suzuki N. (2007). *Coprinellus curtus* (Hitoyo-take) prevents diseases of vegetables caused by pathogenic fungi. *FEMS Microbiol. Lett.* 275 286–291. 10.1111/j.1574-6968.2007.00899.x 17850327

[B52] Oyserman B. O. Cordovez V. Sarango Flores S. W. Nijveen H. Medema M. H. Raaijmakers J. M. (2019). Extracting the GEMs: genotype, Environment and Microbiome interactions shaping host phenotypes. *bioRxiv* [Preprint]. 10.1101/863399 PMC7874016 33584558

[B53] Oyserman B. O. Medema M. H. Raaijmakers J. M. (2018). Road MAPs to engineer host microbiomes. *Curr. Opin. Microbiol.* 43 46–54. 10.1016/j.mib.2017.11.023 29207308

[B54] Pérez-Jaramillo J. E. Carrión V. J. Bosse M. Ferrão L. F. V. De Hollander M. Garcia A. A. F. (2017). Linking rhizosphere microbiome composition of wild and domesticated *Phaseolus vulgaris* to genotypic and root phenotypic traits. *ISME J.* 11 2244–2257. 10.1038/ismej.2017.85 28585939 PMC5607367

[B55] Pfender W. F. Hagedorn D. J. (1982). Comparative virulence of *Aphanomyces euteiches* f. sp. *phaseoli* and *Pythium ultimum* on *Phaseolus vulgaris* at naturally occurring inoculum levels. *Phytopathology* 72 1200–1204. 10.1094/Phyto-72-1200

[B56] Pflughöft O. (2008). *Pilzkrankheiten in Körnerfuttererbsen (Pisum sativum L.) – Diagnose, Epidemiologie, Ertragsrelevanz und Bekämpfung.* Ph.D. dissertation, Universität Göttingen, Göttingen.

[B57] Pflughöft O. Merker C. von Tiedemann A. Schäfer B. C. (2012). Zur Verbreitung und Bedeutung von Pilzkrankheiten in Körnerfuttererbsen (*Pisum sativum* L.) in Deutschland. *Gesunde Pflanz.* 64 39–48. 10.1007/s10343-011-0270-x

[B58] Pilet-Nayel M.-L. Moury B. Caffier V. Montarry J. Kerlan M.-C. Fournet S. (2017). Quantitative resistance to plant pathogens in pyramiding strategies for durable crop protection. *Front. Plant Sci.* 8:1838. 10.3389/fpls.2017.01838 29163575 PMC5664368

[B59] Pilet-Nayel M. L. Muehlbauer F. J. McGee R. J. Kraft J. M. Baranger A. Coyne C. J. (2005). Consistent quantitative trait loci in pea for partial resistance to *Aphanomyces euteiches* isolates from the United States and France. *Phytopathology* 95 1287–1293. 10.1094/PHYTO-95-1287 18943359

[B60] R Core Team, (2018). *R: A Language and Environment for Statistical Computing.* Vienna: R Foundation for Statistical Computing.

[B61] Reckling M. Bergkvist G. Watson C. A. Stoddard F. L. Zander P. M. Walker R. L. (2016). Trade-offs between economic and environmental impacts of introducing legumes into cropping systems. *Front. Plant Sci.* 7:669. 10.3389/fpls.2016.00669 27242870 PMC4876776

[B62] Robinson M. D. McCarthy D. J. Smyth G. K. (2010). edgeR: a Bioconductor package for differential expression analysis of digital gene expression data. *Bioinformatics* 26 139–140. 10.1093/bioinformatics/btp616 19910308 PMC2796818

[B63] Robinson M. D. Oshlack A. (2010). A scaling normalization method for differential expression analysis of RNA-seq data. *Genome Biol.* 11:R25. 10.1186/gb-2010-11-3-r25 20196867 PMC2864565

[B64] Rubiales D. Fondevilla S. Chen W. Gentzbittel L. Higgins T. J. V. V. Castillejo M. A. (2015). Achievements and challenges in legume breeding for pest and disease resistance. *Crit. Rev. Plant Sci.* 34 195–236. 10.1080/07352689.2014.898445

[B65] Rubiales D. Mikic A. (2014). Introduction: legumes in sustainable agriculture. *Crit. Rev. Plant Sci.* 34 2–3. 10.1080/07352689.2014.897896

[B66] Schmieder R. Edwards R. (2011). Quality control and preprocessing of metagenomic datasets. *Bioinformatics* 27 863–864. 10.1093/bioinformatics/btr026 21278185 PMC3051327

[B67] Shehata M. A. Pfleger F. L. Davis D. W. (1983). Response of susceptible and moderately resistant pea genotypes to interaction between *Rhizoctonia solani* and three other stem and root rot pathogens. *Plant Dis.* 67 1146–1148. 10.1094/PD-67-1146

[B68] Šišić A. Baćanović J. Finckh M. R. Sisic A. Bacanovic J. Finckh M. R. (2017). Endophytic Fusarium equiseti stimulates plant growth and reduces root rot disease of pea (*Pisum sativum* L.) caused by *Fusarium avenaceum* and *Peyronellaea pinodella*. *Eur. J. Plant Pathol.* 148 271–282. 10.1007/s10658-016-1086-4

[B69] Šišić A. Bacanovic-Sisic J. Karlovsky P. Wittwer R. Walder F. Campiglia E. (2018). Roots of symptom-free leguminous cover crop and living mulch species harbor diverse Fusarium communities that show highly variable aggressiveness on pea (*Pisum sativum*). *PLoS One* 13:e0191969. 10.1371/journal.pone.0191969 29444142 PMC5812582

[B70] Slezack S. Dumas-Gaudot E. Paynot M. Gianinazzi S. (2000). Is a fully established arbuscular mycorrhizal symbiosis required for bioprotection of *Pisum sativum* roots against *Aphanomyces euteiches*? *Mol. Plant Microbe Interact.* 13 238–241. 10.1094/MPMI.2000.13.2.238 10659715

[B71] Taheri E. A. Chatterton S. Foroud N. A. Gossen B. D. McLaren D. L. Esmaeili Taheri A. (2017). Identification and community dynamics of fungi associated with root, crown, and foot rot of field pea in western Canada. *Eur. J. Plant Pathol.* 147 489–500. 10.1007/s10658-016-1017-4

[B72] Weeden N. McGee R. Grau C. Muehlbauer F. (2000). A gene influencing tolerance to common root rot is located on linkage group IV. *Pisum Genet.* 32 53–55.

[B73] Wei Z. Jousset A. (2017). Plant breeding goes microbial. *Trends Plant Sci.* 22 555–558. 10.1016/j.tplants.2017.05.009 28592368

[B74] White T. J. Bruns T. Lee S. Taylor J. (1990). “Amplification and direct sequencing of fungal ribosomal RNA genes for phylogenetics,” in *PCR Protocols: A Guide to Methods and Applications*, eds Innis M. A. Gelfand D. H. Sninsky J. J. White T. J. (San Diego, CA: Academic Press), 315–322.

[B75] Wille L. Messmer M. M. Studer B. Hohmann P. (2019). Insights to plant-microbe interactions provide opportunities to improve resistance breeding against root diseases in grain legumes. *Plant Cell Environ.* 42 20–40. 10.1111/pce.13214 29645277

[B76] Willsey T. L. Chatterton S. Heynen M. Erickson A. (2018). Detection of interactions between the pea root rot pathogens *Aphanomyces euteiches* and *Fusarium* spp. using a multiplex qPCR assay. *Plant Pathol.* 67 1912–1923. 10.1111/ppa.12895

[B77] Wu L. Chang K.-F. Hwang S.-F. Conner R. Fredua-Agyeman R. Feindel D. (2019). Evaluation of host resistance and fungicide application as tools for the management of root rot of field pea caused by *Aphanomyces euteiches*. *Crop J.* 7 38–48. 10.1016/j.cj.2018.07.005

[B78] Xu L. Ravnskov S. Larsen J. Nicolaisen M. (2012). Linking fungal communities in roots, rhizosphere, and soil to the health status of Pisum sativum. *FEMS Microbiol. Ecol.* 82 736–745. 10.1111/j.1574-6941.2012.01445.x 22775574

[B79] Xue A. G. (2003a). Biological control of pathogens causing root rot complex in field pea using *Clonostachys rosea* Strain ACM941. *Phytopathology* 93 329–335. 10.1094/PHYTO.2003.93.3.329 18944343

[B80] Xue A. G. (2003b). Efficacy of *Clonostachys rosea* strain ACM941 and fungicide seed treatments for controlling the root rot complex of field pea. *Can. J. Plant Sci.* 83 519–524. 10.4141/P02-078

[B81] Yu L. Nicolaisen M. Larsen J. Ravnskov S. (2012a). Molecular characterization of root-associated fungal communities in relation to health status of *Pisum sativum* using barcoded pyrosequencing. *Plant Soil* 357 395–405. 10.1007/s11104-012-1180-0

[B82] Yu L. Nicolaisen M. Larsen J. Ravnskov S. (2012b). Succession of root-associated fungi in *Pisum sativum* during a plant growth cycle as examined by 454 pyrosequencing. *Plant Soil* 358 225–233. 10.1007/s11104-012-1188-5

[B83] Zahid S. Udenigwe C. C. Ata A. Eze M. O. Segstro E. P. Holloway P. (2006). New bioactive natural products from *Coprinus micaceus*. *Nat. Prod. Res.* 20 1283–1289. 10.1080/14786410601101829 17393652

[B84] Zhou X. Lindsay H. Robinson M. D. (2014). Robustly detecting differential expression in RNA sequencing data using observation weights. *Nucleic Acids Res.* 42:e91. 10.1093/nar/gku310 24753412 PMC4066750

