# Supplementary material for: Heritable Variation in Pea for Resistance Against a Root Rot Complex and Its Characterization by Amplicon Sequencing
Source: Front Plant Sci. 2020 Nov 3;11:542153. doi: 10.3389/fpls.2020.542153 (PMC7669989; doi:10.3389/fpls.2020.542153)
Supplement: Supplementary file 1 [file Data_Sheet_1.ZIP › Final_FPSci_submitted_Supinfos_Rev3/ScreenPaper_SUPFig3_C1vsC2.docx]

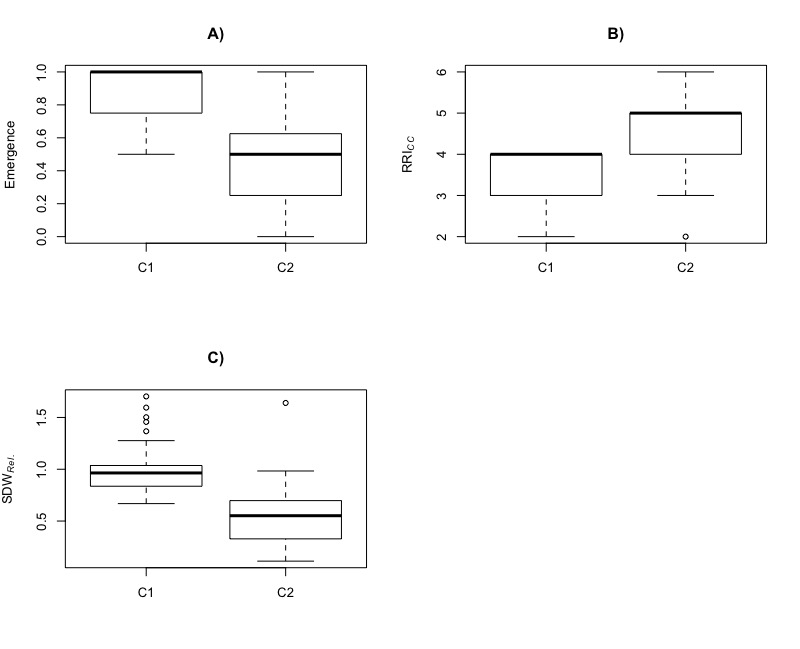


*

*

*

Supplementary Figure 3. Growth characteristics of reference cultivars C1 (cv. 'EFB.33') and C2 (cv. 'Respect') on naturally infested soil. Wilcoxon rank sum test was used to calculate the significance of the difference between the two cultivars (n = 4; means of each replication). A) Emergence rate after 14 days (W= 16, *p*-value = 0.028); B) Root rot index (RRI*_CC_*) after 21 days (W= 16, *p*-value = 0.027); C) Relative shoot dry weight (SDW*_Rel._*) after 21 days (W= 16, *p*-value = 0.029).
