# Supplementary material for: Heritable Variation in Pea for Resistance Against a Root Rot Complex and Its Characterization by Amplicon Sequencing
Source: Front Plant Sci. 2020 Nov 3;11:542153. doi: 10.3389/fpls.2020.542153 (PMC7669989; doi:10.3389/fpls.2020.542153)
Supplement: Supplementary file 1 [file Data_Sheet_1.ZIP › Final_FPSci_submitted_Supinfos_Rev3/ScreenPaper_SUPFig1_SvsNS.docx]

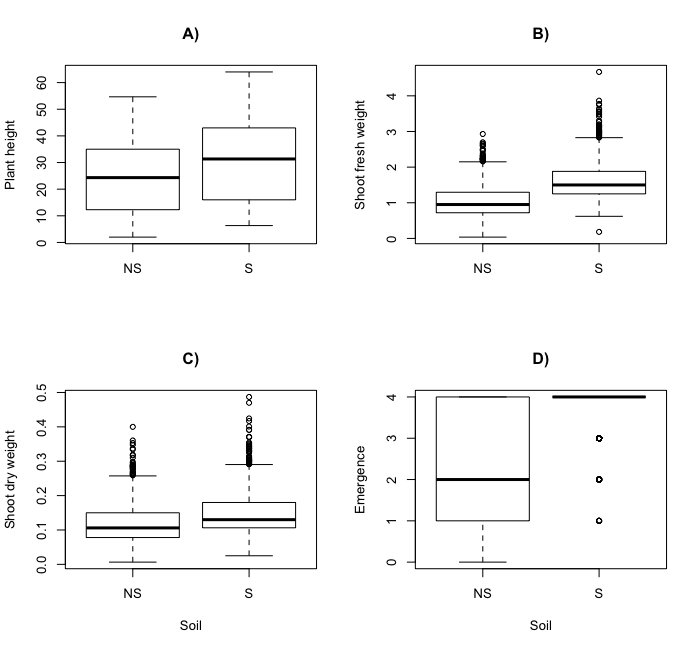


***

***

***

***

Supplementary Figure 1. Growth characteristics of 261 pea lines in naturally infested field soil (NS) and in sterilised soil (S). Paired t-tests were used to calculate the significance of the difference between the treatment means. For emergence a Wilcoxon signed rank test was applied. A) Overall mean plant height after 21 days (t = 29.15, df = 982, *p*-value < 0.001); B) overall mean shoot fresh weight after 21 days (t = 39.7, df = 980, *p*-value < 0.001); C) overall mean shoot dry weight after 21 days (t = 18.5, df = 975, *p*-value < 0.001); D) overall mean plant emergence after 14 days (V = 313710, *p*-value < 0.001).
