# Supplementary material for: Heritable Variation in Pea for Resistance Against a Root Rot Complex and Its Characterization by Amplicon Sequencing
Source: Front Plant Sci. 2020 Nov 3;11:542153. doi: 10.3389/fpls.2020.542153 (PMC7669989; doi:10.3389/fpls.2020.542153)
Supplement: Supplementary file 1 [file Data_Sheet_1.ZIP › Final_FPSci_submitted_Supinfos_Rev3/ScreenPaper_SUPTab2_Soilproperties.docx]

Supplementary Table 2. Soil characteristics of two field sites with strong signs of pea root rot complex (Fuchs et al., 2014). Soil from site ‘heavy infestation’ was used for the controlled conditions resistance screening of 261 pea lines. In 2018, a selection of nine lines was evaluated in a field trial on both sites. Soil samples were taken on 14 March 2018 with a soil auger (3 cm diameter) to 20 cm depth in each plot. Twenty-four samples per site were pooled and homogenised before analysis. Grain composition, organic matter content (OM), pH and soil nutrient analysis (except N_min_) were performed by the *Labor für Boden- und Umweltanalytik* (lbu), Switzerland. N and C analysis were performed by the *Research Institute for Organic Agriculture* (FiBL), Switzerland according to Agroscope (1996).

|  |  | | |  |  | | |  |  |  | | | | | | | |
| --- | --- | --- | --- | --- | --- | --- | --- | --- | --- | --- | --- | --- | --- | --- | --- | --- | --- |
|  | Grain composition | | |  | Soil nutrient content [%] | | |  |  | Soil nutrient content [mg kg^-1^] | | | | | | | |
| Site | Clay | Silt | Sand | pH | N_tot_ | C_tot_ | C_org_ |  |  | N_min_ | P | K | Mg | B | Mn | Cu | Fe |
| ‘Heavy infestation’ | 16.5 | 37.4 | 46.1 | 7.1 | 0.19 | 1.95 | 1.90 |  |  | 2.6 | 123.4 | 115.9 | 90.3 | 0.6 | 179 | 12.2 | 253 |
| ‘Moderate infestation’ | 16.5 | 28.9 | 54.6 | 6.8 | 0.19 | 1.91 | 1.89 |  |  | 3.9 | 65.1 | 106.6 | 120.9 | 0.4 | 263 | 13.3 | 270 |

Agroscope (1996). *Schweizerische Referenzmethoden der Eidg. landwirtschaftlichen Forschungsanstalten.* Zürich-Reckenholz: Eidg. Forschungsanstalt für Landwirtschaftlichen Pflanzenbau, FAP.

Fuchs, J.G., Thuerig, B., Brandhuber, R., Bruns, C., Finckh, M.R., Fliessbach, A., Mäder, P., Schmidt, H., Vogt-Kaute, W., Wilbois, K.P., and Lucius, T. (2014). Evaluation of the causes of legume yield depression syndrome using an improved diagnostic tool. *Applied Soil Ecology* 79**,** 26-36.
