# Supplementary material for: Heritable Variation in Pea for Resistance Against a Root Rot Complex and Its Characterization by Amplicon Sequencing
Source: Front Plant Sci. 2020 Nov 3;11:542153. doi: 10.3389/fpls.2020.542153 (PMC7669989; doi:10.3389/fpls.2020.542153)
Supplement: Supplementary file 1 [file Data_Sheet_1.ZIP › Final_FPSci_submitted_Supinfos_Rev3/ScreenPaper_SUPFig4_PairPlot11Estims.docx]

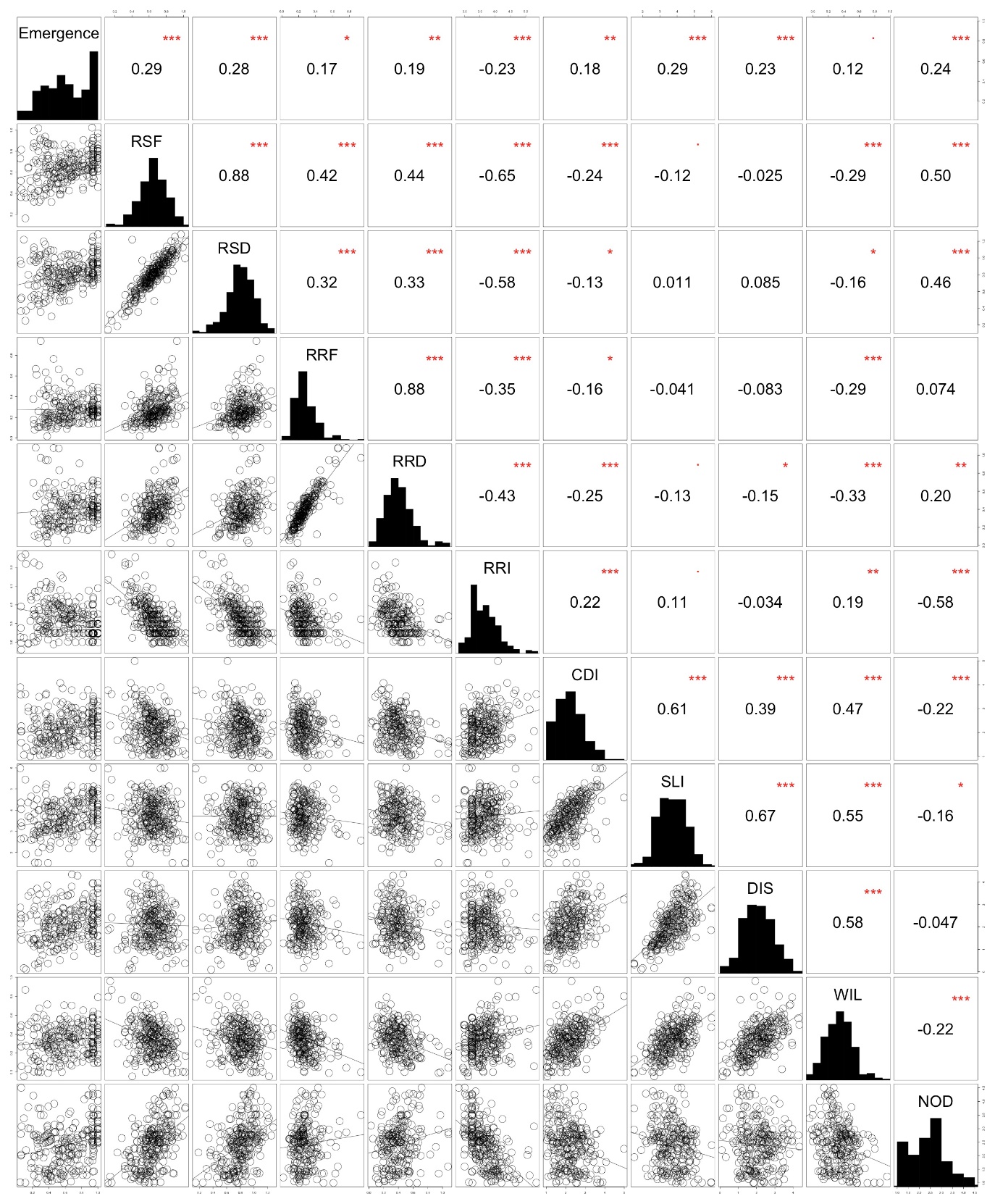


SFW*_Rel._*

SDW*_Rel._*

RFW*_Rel._*

RDW*_Rel._*

Supplementary Figure 4. Pair plot of mixed model estimated genotypic means for eleven traits (Emergence = emergence rate; RFW*_Rel._*, RDW*_Rel._*, SFW*_Rel._*, and SDW*_Rel._* = relative root and shoot fresh and dry weight, respectively; NOD = nodulation index; RRI = root rot index; WIL = wilted nodes; CDI = cortex decay index; SLI = shoot lesion index; DIS = disease progress; see Material and Methods for a detailed description). Panels on the diagonal show the distribution of the genotypic means for each trait. Panels below the diagonal show pairwise correlations with a linear regression line. Panels above the diagonal display correlation coefficients (Spearman's rho) and associated significance levels (^•^ = *p*-value < 0.1; * = < 0.5; ** = < 0.01; *** = < 0.001).
