# Supplementary material for: Heritable Variation in Pea for Resistance Against a Root Rot Complex and Its Characterization by Amplicon Sequencing
Source: Front Plant Sci. 2020 Nov 3;11:542153. doi: 10.3389/fpls.2020.542153 (PMC7669989; doi:10.3389/fpls.2020.542153)
Supplement: Supplementary file 1 [file Data_Sheet_1.ZIP › Final_FPSci_submitted_Supinfos_Rev3/ScreenPaper_SUPFig2_RRI.docx]

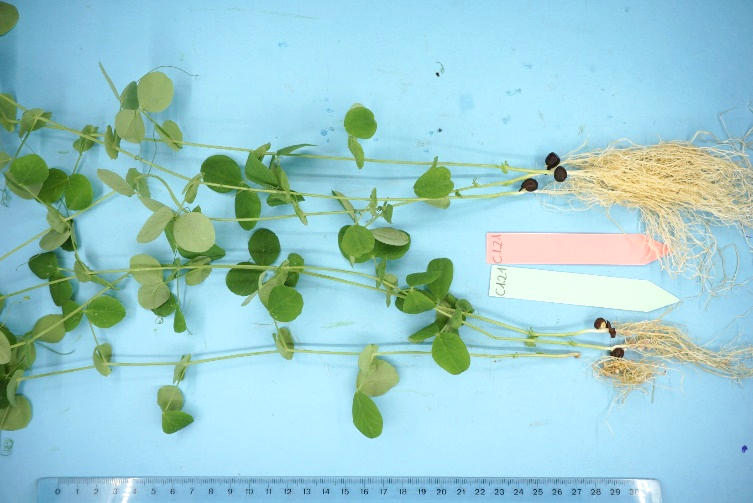

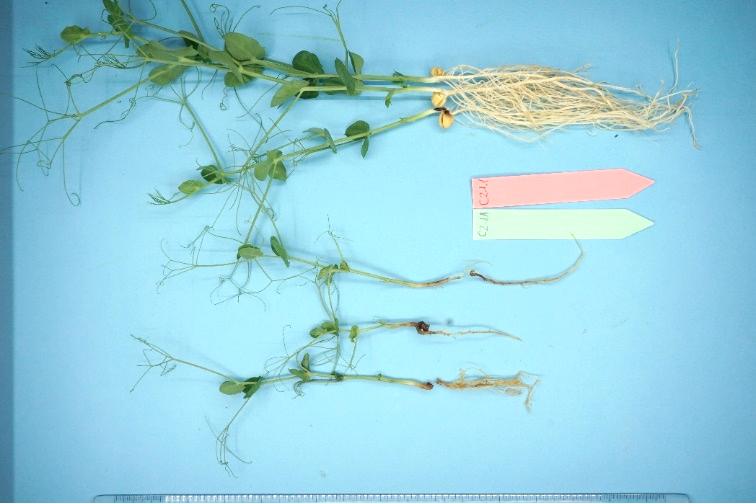

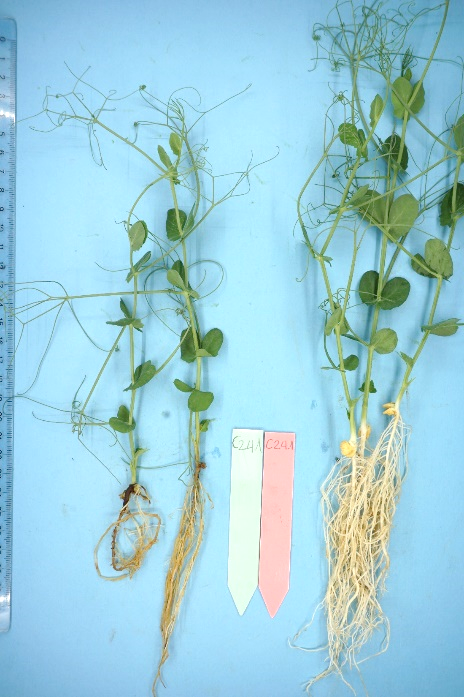


**2**

**4**

**6**

**A**

**C**

**E**

**1**


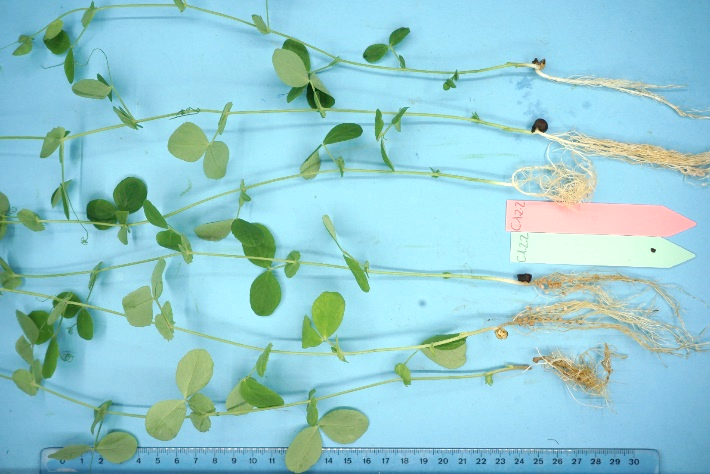

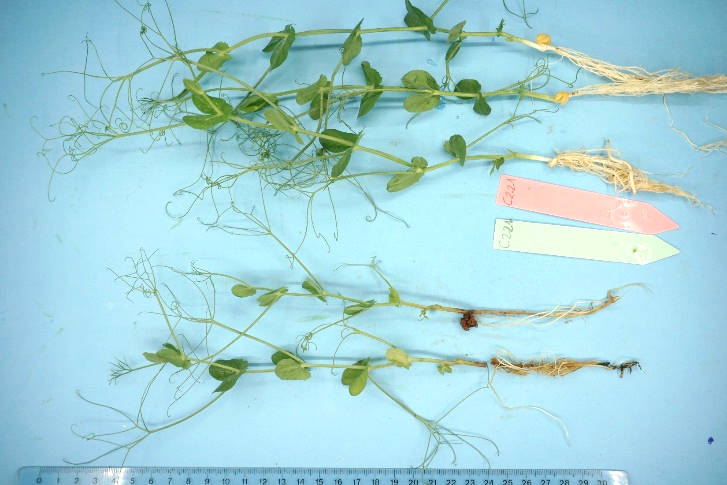


**D**

**B**

**3**

**5**

Supplementary Figure 2. Root rot index (RRI*_CC_*) on a 1 to 6 scoring scale (1 = no symptoms, 2 = small localised lesions on lower stem or upper root, covering less than 50% of circumference, 3 = light brown discoloration and moderate disintegration (< 30% compared to uninoculated control) of the root system, 4 = dark brown discoloration and strong disintegration (> 30% compared to uninoculated control) of the root system, 5 = only tap root left attached to the plant, 6 = complete disintegration of the root system). Plants grown on infested field soil are on the left side in the picture, plants grown on sterilized field soil on the right. The levels 1 – 6 are displayed in the pictures. Pictures (A) and (B) display pea genotype C1 (cv. ‘EFB.33’). Pictures (C), (D) and (E) display pea genotype C2 (cv. ‘Respect’). A 30 cm ruler is included in each picture.
