# Supplementary material for: Heritable Variation in Pea for Resistance Against a Root Rot Complex and Its Characterization by Amplicon Sequencing
Source: Front Plant Sci. 2020 Nov 3;11:542153. doi: 10.3389/fpls.2020.542153 (PMC7669989; doi:10.3389/fpls.2020.542153)
Supplement: Supplementary file 1 [file Data_Sheet_1.ZIP › Final_FPSci_submitted_Supinfos_Rev3/ScreenPaper_SUPFig5_NGSrarefaction.docx]

Sample size (sequences)

Number of OTUs


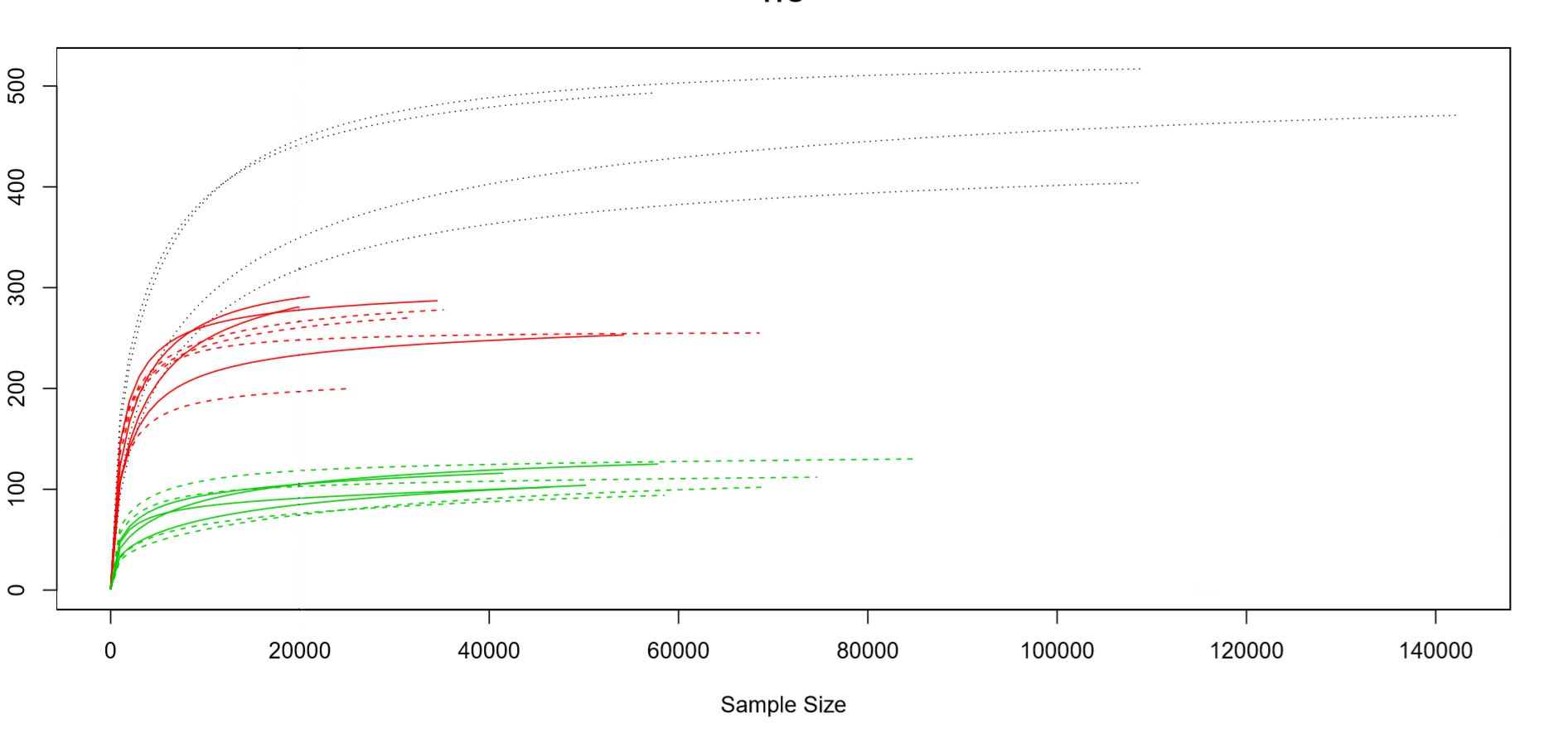


Supplementary Figure 4. Rarefaction curves of the ITS amplicon sequencing data of bulk soil (n = 4; grey), rhizosphere soil (n = 8; red) and root (n = 8; green) samples. For the rhizosphere and root samples dashed and solid lines represent pea lines S164 and S12, respectively. OTUs were defined at a 97% similarity threshold.
