# Supplementary material for: Heritable Variation in Pea for Resistance Against a Root Rot Complex and Its Characterization by Amplicon Sequencing
Source: Front Plant Sci. 2020 Nov 3;11:542153. doi: 10.3389/fpls.2020.542153 (PMC7669989; doi:10.3389/fpls.2020.542153)
Supplement: Supplementary file 1 [file Data_Sheet_1.ZIP › Final_FPSci_submitted_Supinfos_Rev3/ScreenPaper_SUPTab3_OrdiTraits.docx]

Supplementary Table 3. Overview of four ordinal variables assessed on pea lines in the controlled conditions resistance screening. Plants were grown on naturally infested field soil in four replications and evaluated 21 days after sowing.

|  | Level | | | | | | |
| --- | --- | --- | --- | --- | --- | --- | --- |
|  | 1 | 2 | 3 | 4 | 5 | 6 | 7 |
| RRI*_CC_* | no symptoms | small localised lesions on lower stem or upper root, covering less than 50% of circumference | light brown discoloration and moderate disintegration  (< 30% compared to uninoculated control) of the root system | dark brown discoloration and strong disintegration  (> 30% compared to uninoculated control) of the root system | only tap root left attached to the plant | complete disintegration of the root system | NA |
| CDI | no symptoms | cortex locally cracked | local disintegration of the cortex  (< 5mm) | strong disintegration of the cortex (>5mm) and vascular tissue visible | total disintegration of cortex, roots hanging attached to vascular tissue | NA | NA |
| SLI | no symptoms | small localised discolorations | spread of discoloration up to max. second lowest shoot node | spread of discoloration above second node, | discoloration and drying-out or soft, water-soaked stem base | discoloration and disintegration of stem base | NA |
| NOD | no nodules | 1-5 nodules | 6-10 nodules | 11-20 nodules | 21-40 nodules | 41-60 nodules | 7 > 60 nodules |
